# Building a Green, Robust, and Efficient Bi-MOF Heterogeneous Catalyst for the Strecker Reaction of Ketones

**DOI:** 10.1021/acs.inorgchem.2c00628

**Published:** 2022-05-05

**Authors:** Eloy P. Gómez-Oliveira, Nayara Méndez, Marta Iglesias, Enrique Gutiérrez-Puebla, Lina M. Aguirre-Díaz, M. Ángeles Monge

**Affiliations:** Departamento de Nuevas Arquitecturas en Química de Materiales, Instituto de Ciencia de Materiales de Madrid, Consejo Superior de Investigaciones Científicas, Sor Juana Inés de la Cruz 3, Cantoblanco, Madrid 28049, Spain

## Abstract

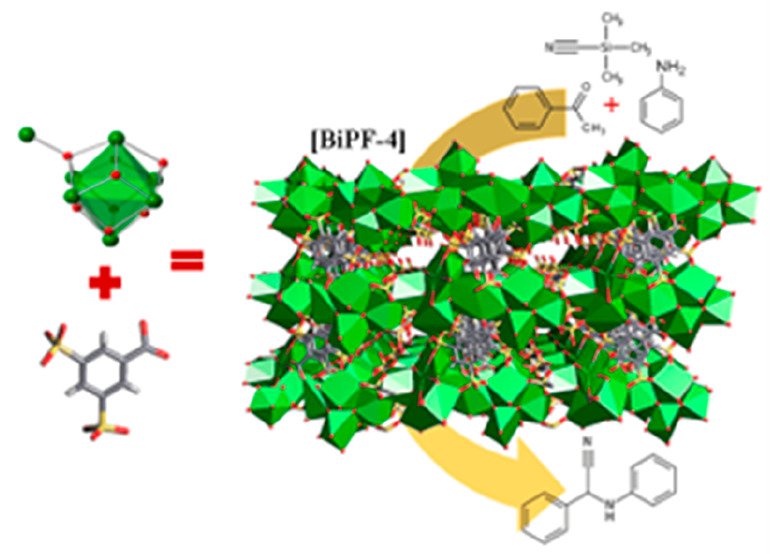

In this work, we present the new **[Bi_14_(μ_3_-O)_9_(μ_4_-O)_2_(μ_3–_OH)_5_(3,5-DSB)_5_(H_2_O)_3_]·7H_2_O**, **BiPF-4** (bismuth polymeric framework—4) MOF, its microwave hydrothermal synthesis, as well as its behavior as a heterogeneous catalyst in the multicomponent organic Strecker reaction. The **BiPF-4** material shows a three-dimensional (3D) framework formed by peculiar inorganic oxo-hydroxo-bismutate layers connected among them through the 3,5-dsb (3,5-disulfobenzoic acid) linker. These two-dimensional (2D) layers, built by junctions of Bi7 polyhedra SBU, provide the material of many Lewis acid catalytic sites because of the mixing in the metal coordination number. **BiPF-4** is a highly robust, green, and stable material that demonstrates an excellent heterogeneous catalytic activity in the multicomponent Strecker reaction of ketones carried out in one-pot synthesis, bringing a reliable platform of novel green materials based on nontoxic and abundant metal sources such as bismuth.

## Abstract 2

In this work, we present the new **[Bi_14_(μ_3_-O)_9_(μ_4_-O)_2_(μ_3−_OH)_5_(3,5-DSB)_5_(H_2_O)_3_]·7H_2_O**, **BiPF-4** (bismuth polymeric framework—4) MOF, its microwave hydrothermal synthesis, as well as its behavior as a heterogeneous catalyst in the multicomponent organic Strecker reaction.

## Introduction

Within the increasing environmental emergency that our society is facing, the development of functional materials, which can be prepared by the following low environmental impact processes based on the use of nontoxic elements, is of great significance. Over the years, bismuth-based processes have been developed and applied based on their unusually low toxicity. Thus, bismuth compounds are usually accepted as biologically safe and nontoxic materials^1,2^ and have found wide application as antibacterial and antifungal agents,^3−6^ for example, bismuth sulfonates that are effective against *H. pylori* even when compared to commercial drugs.^7^ In solid-state chemistry, bismuth compounds have been studied in important applications including high TC superconductors,^8^ ferroelectric and piezoelectric materials,^9^ oxide ion conductors,^10,11^ catalysis,^12−16^ thermoelectric materials,^17^ among others. In the view of this, bismuth appears as an excellent candidate to be used in the construction of metal–organic frameworks (MOFs) because they and their derivatives are a class of emerging promising coordination materials. There have been several facts that prevented from a quick development of the Bi-MOFs, namely, (i) flexible coordination geometry of Bi^3+^, which attributes to the lone pair of a hemi-directed coordination polyhedra tendency leading to compact one-dimensional (1D) and two-dimensional (2D) frameworks, (ii) tendency of Bi^3+^ to form anionic frameworks as Bi has up to 10 coordination sites, (iii) hydrolysis reactivity of Bi^3+^ to form oxides and hydroxides, and (iv) limited solubility of Bi^3+^ salts. In spite of these facts, in the last decade, an increasing number of new Bi-MOFs with different SBUs^7,18−29^ have appeared, some of them showing outstanding potential in multiple fields.^30^

As for the linker, sulfonic acids with p*K*_a_ values generally in the range of 1–3 are much stronger acids than their carboxylic acid analogues (p*K*_a_ ≈ 3–5), and hence, they create an ideal environment for the formation of oxo/oxo-hydroxo-bismutate clusters in the aqueous solution and the presence of water,^31^ which can be introduced as MOF SBUs. Thus, sulfonate-carboxylate linkers have already been successfully employed to synthesize a few bismuth MOFs with different types of inorganic SBUs, highlighting the structural richness that the use of this element offers for accessing new structures.^32,33^ 3,5-disulfobenzoic acid (H_3_DSB) is another tritopic organic linker combining carboxylic and sulfonic acid coordinating groups, which we have previously employed in the formation of several rare-earth MOFs, showing various types of polynuclear inorganic SBUs.^34−36^

With this context, following our interest in the study of the influence of [μ-OH] and [μ = O] species on the structure formation, and the development of novel MOFs as heterogeneous Lewis acid catalysts,^37^ we have now combined bismuth and H_3_DSB to prepare a new MOF with catalytic activity for the multicomponent Strecker reaction with ketones. The Strecker reaction is a versatile way of preparing α-aminonitriles through the attack of a nitrile group to an imine group. The resulting α-aminonitriles can be hydrolyzed to obtain α-amino acids or used as intermediates in the preparation of nitrogen-containing heterocycles (such as imidazoles and thiadiazoles) that are significant in organic chemistry.^38^

**Scheme 1:**
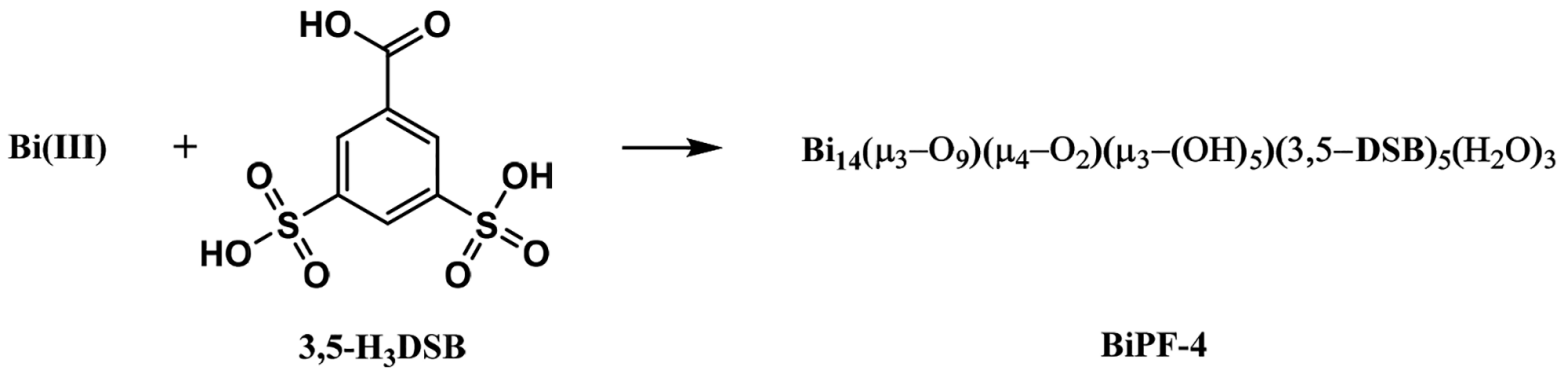
Material **BiPF-4** is Obtained via the Hydrothermal Reaction of Bismuth Nitrate with the Sodium Salt of 3,5-DSB3-Linker under Microwave Heating

The new compound, denoted as **BiPF-4** (bismuth polymeric framework—4), has been synthesized under hydrothermal conditions, with water as a unique solvent and the use of microwave heating during short reaction times (1 h) (Scheme 1).

The crystal structure of **BiPF-4** includes inorganic SBUs built of seven bismuth atoms with different coordination environments, which are joined together to create a 3D, highly robust framework. Thus, the material is chemically and thermally stable, and it exhibits high activity and selectivity as a heterogeneous catalyst in the three-component Strecker reaction of ketones carried out in one-pot synthesis, highlighting the potential to access new Lewis acid catalyst with the use of a nontoxic and earth-abundant element such as bismuth.

## Experimental Section

### General Information

All reagents and solvents employed were commercially available and used as supplied without further purification: 3,5-disulfobenzoic acid, disodium salt (98% Sigma-Aldrich), and bismuth nitrate pentahydrate (99.9% Strem Chemicals). The infrared (IR) spectra were recorded from KBr pellets in the range 4000–250 cm^–1^ on a Bruker IFS 66 V/S. The thermogravimetric and differential thermal analyses (TGA–DTA) were performed using a Seiko TG/DTA 320U equipment in a temperature range between 25 and 1000 °C in air (100 mL/min flow) atmosphere and a heating rate of 10 °C/min. A Perkin-Elmer CNHS Analyzer 2400 was employed for the elemental analysis. Powder X-ray diffraction (PXRD) patterns were measured with a Bruker D8 diffractometer, with step size = 0.02° and exposure time = 0.5 s/step.

### Synthesis and Characterization

The compound was synthesized under microwave heating at 180 °C for one hour. The molar composition of the initial reaction mixture was Bi(NO_3_)·5H_2_O (100 mg, 0.21 mmol) + 3,5-HDSBNa_2_ (67 mg, 0.24 mmol) + water 4 mL. This clear solution was placed in a 10 mL vessel, and after the heating time, the obtained white crystals were washed with water, ethanol (2×), and acetone (5 mL each). The solid was then immersed in acetone (5 mL), which was freshly exchanged twice a day for 3 days, and subsequently dried in air. Elemental analysis results 8.83% C, 1.02% H y 6.58% S (calculated: 8.81% C, 1.08% H, 6.72% S for C_35_H_51_Bi_14_O_66_S_10_ = **[Bi_14_(μ_3_-O)_9_(μ_4_-O)_2_(μ_3–_OH)_5_ (3,5-DSB)_5_(H_2_O)_3_]·7H_2_O, BiPF-4** IR (KBr, cm^–1^): 3434 ν(O–H), 1602 ν(C=O), 1536 ν(C–O), 1363 ν(C–S), 1201, 1112, 1033, 639, and 610 ν(C–C) aromatic, 784 ν(C–C), 519, and 431 ν(Bi–O), Figure S1. PXRD analysis confirms the phase purity of the bulk sample, and **BiPF-4** is stable in air, with no changes in the PXRD pattern after several weeks exposed to air (Figure S2) and different pH, Figure S3. TGA shows that **BiPF-4** is thermally stable. After an initial weight loss at low temperature (<100 °C) attributable to adsorbed water molecules, the material shows thermal stability up to above 400 °C (Figures S4, S5). Residues of TGA at 800 °C are Bi metal in N_2_ and a mixture of Bi_14_(SO_4_)_5_O_16_ and Bi_8_(SO_4_)_3_O_9_ in air (Figures S6 and S7).

### Catalytic Reaction Procedure

A mixture of ketone, amine, and trimethylsilyl cyanide (TMSCN) was added to a Schlenk tube (1.89 mmol ketone, 1.89 mmol amine, and 1.98 mmol TMSCN), where the MOF has been previously introduced. The mixture (in solvent-free conditions) was stirred (300 rpm) at 25 °C between 2.5 and 24 h, depending on the amount of the catalyst, under a N_2_ atmosphere. For comparative purposes, the same standard reaction conditions were used for all tested substrates, and therefore, the reaction parameters were not further optimized for each one of them. The completion of the reaction was monitored by ^1^H-NMR. When the reaction was completed and samples were taken, the content of the Schlenk tube was mixed with DCM in order to dissolve the Strecker α-aminonitrile and recover the catalyst by centrifugation. XRD patterns were obtained before and after each experiment to ensure crystallinity, purity (Figure S8), and recyclability of the MOF (Figure S9).

## Results and Discussion

The crystal structure was determined using single-crystal X-ray diffraction. Details of data collection, refinement, and crystallographic data for **BiPF-4** are summarized in Table S1.

**Figure 1:**
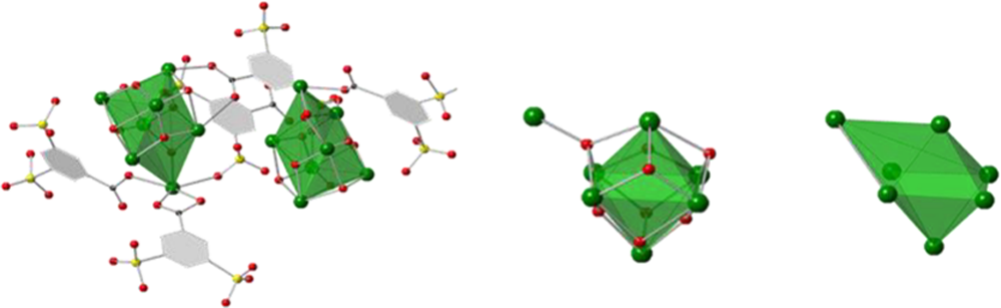
Ball-and-stick view of the asymmetric unit of BiPF-4, including five DBS organic linkers and two Bi7 oxo-hydroxo clusters. Bi is green, C is gray, O is red, and S is yellow.

**BiPF-4** material, formula **[Bi_14_(μ_3_-O)_9_(μ_4_-O)_2_(μ_3_–OH)_5_ (3,5-DSB)_5_(H_2_O)_3_]·7H_2_O**, crystallizes in the triclinic system, with *a* = 10.915(14) Å, *b* = 11.108(12) Å, *c* = 17.648(18) Å, α = 90.59(3)°, β = 98.45(4)°, and γ = 104.26(4)°. Although the structure could be solved in the *P*1̅ space group, a heavy disorder coming from one of the 3,5-DSB linkers made evident the existence of pseudo-symmetry. The Bi cation positions correspond to the centrosymmetric *P*1̅ group, while one of the sulfonate linkers breaks this symmetry. Subsequently, the structure could be solved and refined in the *P*1 space group with the expected pseudo-symmetry correlation problems. Therefore, the acentric asymmetric unit consists of 14 Bi^+3^ ions, five fully deprotonated 3,5-dsb linker cations, nine oxygen atoms in a μ_3_-oxide disposition, two oxygen atoms in a μ_4_-oxide disposition, μ_3_-five hydroxyl oxygen atoms, and three coordinated water molecules (Figure 1).

**Scheme 2:**
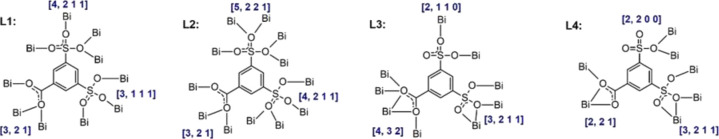
Different Binding Modes of the 3,5-DSB Linker Observed in the BiPF-4 Material For a better understanding the Harris notation is presented for each carboxylate and sulfonate group.

In order to describe the binding mode of the sulfonate and carboxylate groups for all the five crystallography independent linkers, the Harris notation^39^ is used and shown in Scheme 2.

The position of the hydrogen atoms could not be determined from the electron density maps, consequently, the binding modes, the Bi–O distances, and the formula electric neutrality were used to assign the oxygen atoms to water molecules, hydroxyl groups, or O^2–^ anions. The coordinated terminal three water molecules exhibit larger Bi–O distances (2.888, 2.924, and 2.729 Å) than the bridging μ_3_-O^2–^and μ_3_-OH^–^ ions, which are within the 2.076–2.492 Å range. In the case of the two μ_4_-O^2–^ ions, Bi–O are in the 2.178–2.904 Å range, which agrees with the distances reported for other Bi–O systems showing large [Bi_x_O_y_] clusters in coordination polymers.^40^

**Figure 2:**
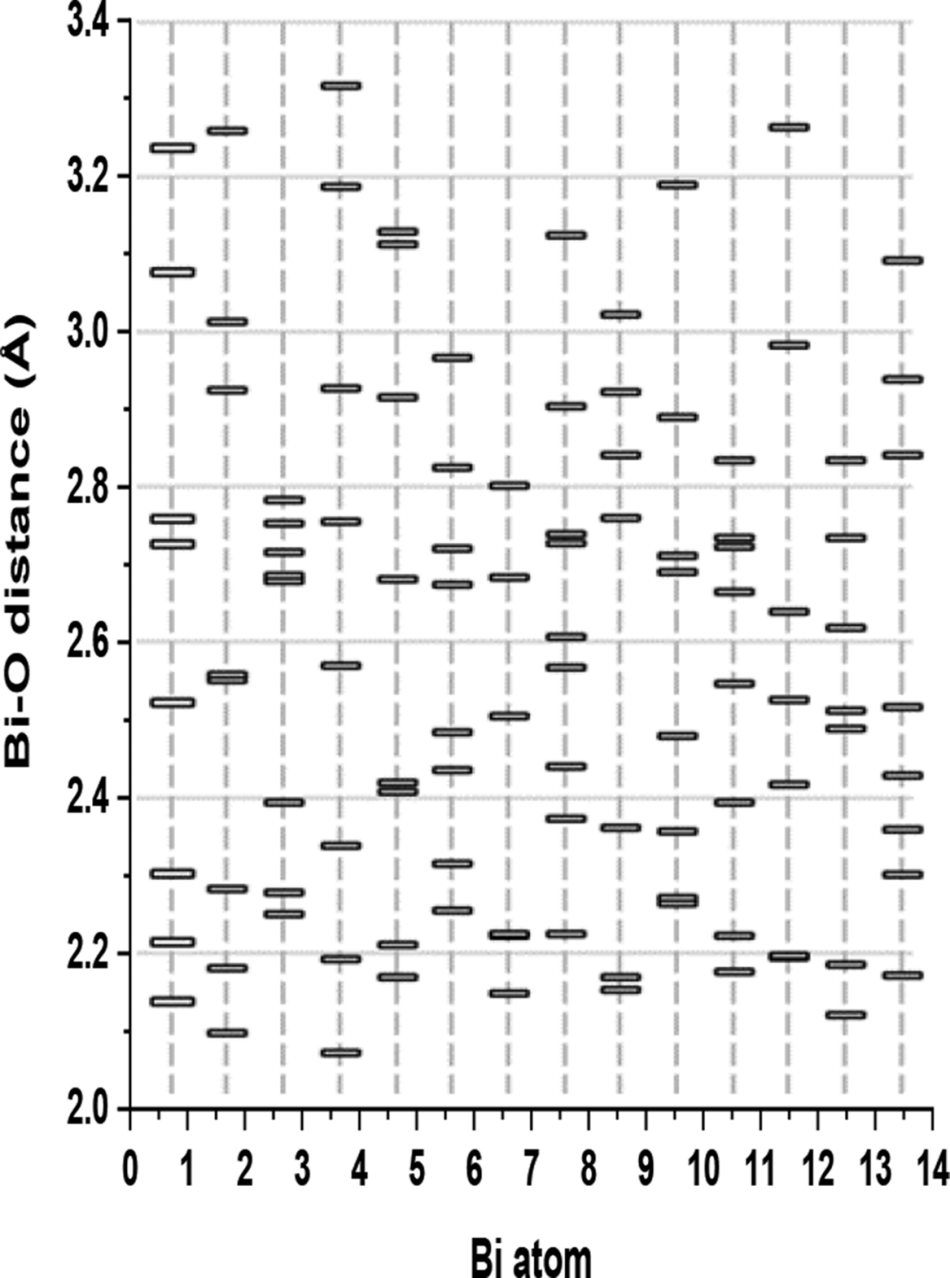
Distribution of the Bi–O distances crystallographically determined for the 14 Bi atoms in **BiPF-4**.

Based on these considerations, we could determine different Bi^3+^ coordination numbers for the 14 bismuth cations of the asymmetric unit. One out of them is coordinated to nine oxygen atoms CN = 9, nine are octacoordinated CN = 8, three have a CN = 7, and the last one is bonded only to six oxygen atoms CN = 6 (Figure 2). This arrangement gives rise to two slightly different SBUs. Although the seven Bi atoms in each Bi7 cluster are equally disposed in both SBUs, (the structure would be centrosymmetric for the Bi cations), the different binding modes of the 3,5-DSB linker avoid the symmetry in the unit cell and force some Bi cations not to have the same CN in both SBUs.

**Figure 3:**
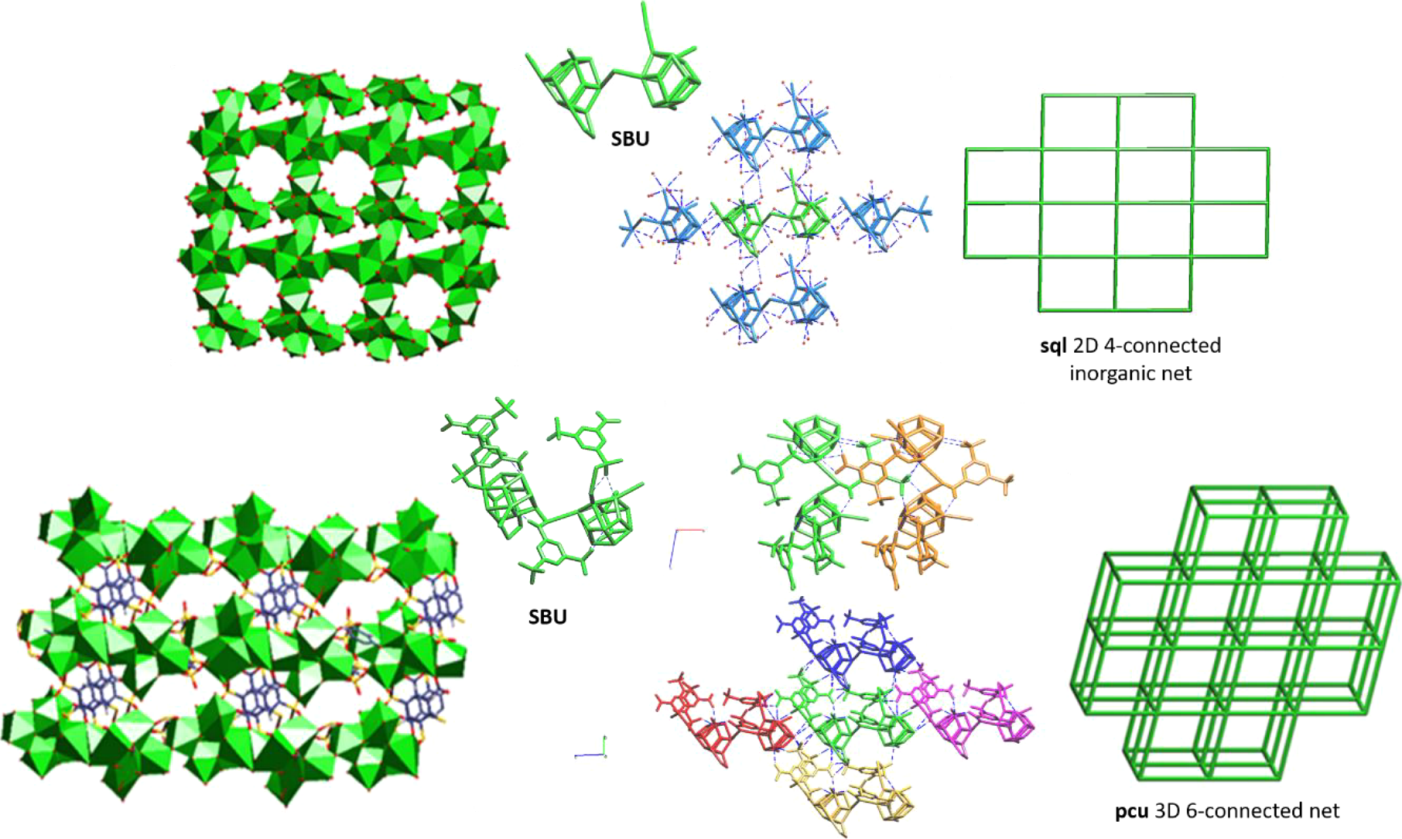
Above: polyhedral representation of the [Bi_14_O_27_] layers perpendicular to the [100] and its corresponding topological **sql** representation of the four-connected 2D inorganic layer, considering the actual no centrosymmetric Bi14 SBU. Down: polyhedral representation of the joint through the organic part of the inorganic layers and the SBU selected to build the topological **pcu** representation of the six-connected 3D net.

Six out of the Bi7 clusters are octahedral-disposed, in a way similar to the well-known M_6_(OH)_4_O_4_ oxo-hydroxo cluster commonly found in zirconium or rare-earth^41,42^ MOFs. In the present case, one of the μ_3_-OH^–^ groups behaves as a μ_4_-O^2–^ unit, coordinating to the seventh bismuth atom (Figure 1). This building unit has already been observed in other materials combining bismuth and sulfonate-carboxylate linkers.^32^ Joints of these SBUs through sulfonate or carboxylate oxygen atoms give rise to [Bi_14_O_27_] layers, which are perpendicular to the [100] direction in a **sql** topological disposition. These oxo-hydroxo-bismutate inorganic layers are connected through the organic part to build a 3D framework (Figure 3).

**Figure 4:**
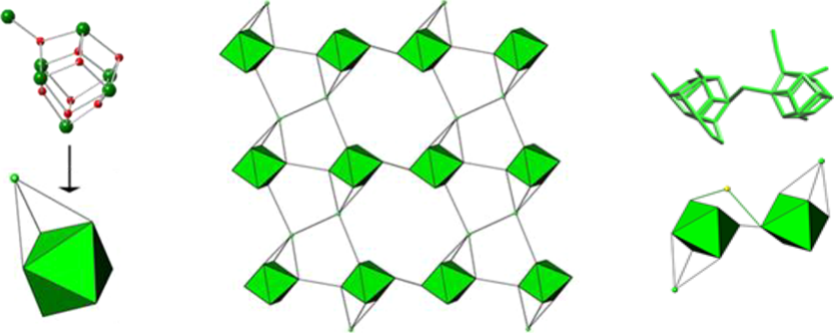
From left to right: representation of the Bi7 clusters as monocaped octahedron with the seventh Bi atom as the green sphere, centrosymmetric Bi arrangement in the inorganic layer, and actual acentric Bi14 SBU.

**Figure 5:**
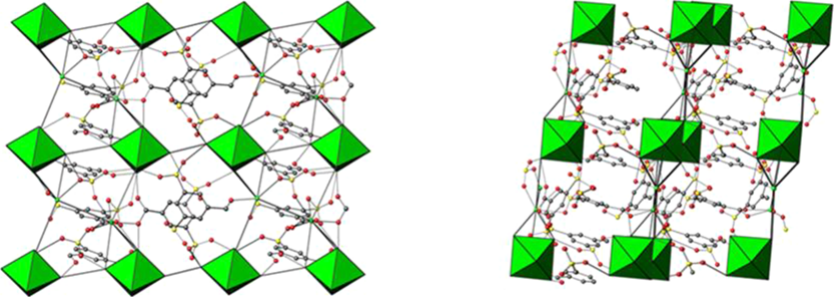
View of the linker arrangement in the layer built by Bi7 clusters left) perpendicular to the [100] and right) along [010] directions.

Looking only at the Bi cation arrangement shown in Figure 4, the Bi7 clusters form mono-capped octahedra, which are linked among them through sulfonate or carboxylate bridges. This arrangement gives rise to Bi inorganic layers perpendicular to the [100] direction. There are three Bi–Bi ranges of distances that involved in the Bi_6_ oxo-hydroxy octahedra (3.73–3.80), those belonging to the Bi_4_ tetrahedra of each monocaped Bi_7_ clusters (4.02–4.15), and finally, the longest ones, due to the joint of adjacent Bi_7_ SBUs through sulfonate or carboxylate bridges (5.04 to 5.23). The connection of the layers through the organic linker along the [100] direction results in the formation of a 3D framework that displays a **pcu** topology net; Figure 3 (down). A view of the linker arrangement in the layer built by Bi7 clusters is shown in Figure 5.

Catalytic activity of **BiPF-4** material in the one-pot Strecker reaction with ketones.

In our previous studies, we have demonstrated that several MOF catalysts containing indium as the metal center were able to conduct in a green and efficient way acid-catalyzed carbon–carbon bond formation in multicomponent reactions using aldehydes as the carbonyl source.^27,37−39^ When ketones are used as carbonyl reactants, typically lower conversion rates are achieved, and stronger conditions such as higher temperatures and extended reaction times are required, and only a few heterogeneous catalysts have shown good conversions (Tables S2 and S3). Among them, some indium-based MOFs have shown remarkably high activity.^39^ In the case of bismuth, a few MOFs have been reported exhibiting catalytic activity, and their Lewis acid character has been tested mainly in reactions such as the ring-opening of styrene oxide to 2-methoxy-2-phenylethanol.^43^

In view of the high Lewis acid character potential exhibited by material **BiPF-4**, we evaluated its catalytic activity by using the multicomponent Strecker organic transformation with the use of ketones. **BiPF-4** exhibits several structural features that could boost its catalytic activity in this reaction: on the one hand, the Bi^+3^ variable coordination number (from 6 to 9) in the SBU and the removable coordinated water molecules favor the accessibility of the reactants to the cluster acid sites. On the other, the presence of OH^–^ groups, and of some noncoordinated S–O oxygen atoms, seems to be decisive factors to create a two-component catalytic system, based on the “dual activation” phenomenon. These facts make **BiPF-4** a promising compound to behave as a dual catalyst.

Therefore, we started by testing the catalytic activity of the **BiPF-4** in the one-pot Strecker reaction, with a screening of catalyst loadings, scaled up, and leaching in the use of acetophenone, aniline, and TMSCN (1:1:1.1) (Table S4). Catalytic amounts of the MOF were placed in a Schlenk tube, followed by the addition of three reactants. The reactions were performed without a solvent at room temperature. Catalyst purity was checked by PXRD (Figure S8). The results of the reactions are summarized in Table 1. Under the selected conditions, a high conversion was achieved (95% yield) to almost exclusively produce the α-aminonitrile product (98% selectivity) (Table 1, entry1). Good yield and selectivity values were also obtained with para-methyl acetophenone (Table 1, entry 2), and excellent conversion and selectivity were observed for a linear aliphatic ketone (Table 1, entry 5), and only in the case of cyclohexanone, the reaction failed to proceed to the formation of amino nitrile, and exclusively, the imine product was observed (Table 1, entry 4). Similarly, when using 4-chloroaniline, or the aliphatic butylamine, excellent yield and conversion values were obtained, and only in the case of 4-methoxyaniline, the yield obtained was low. It should be noted that because the material is not porous, the catalysis is probably taking place on the surface.

**Table 1:**
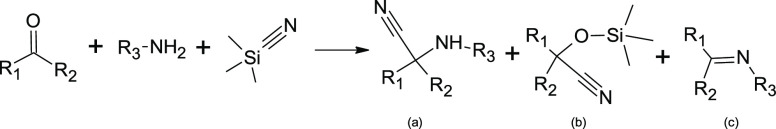

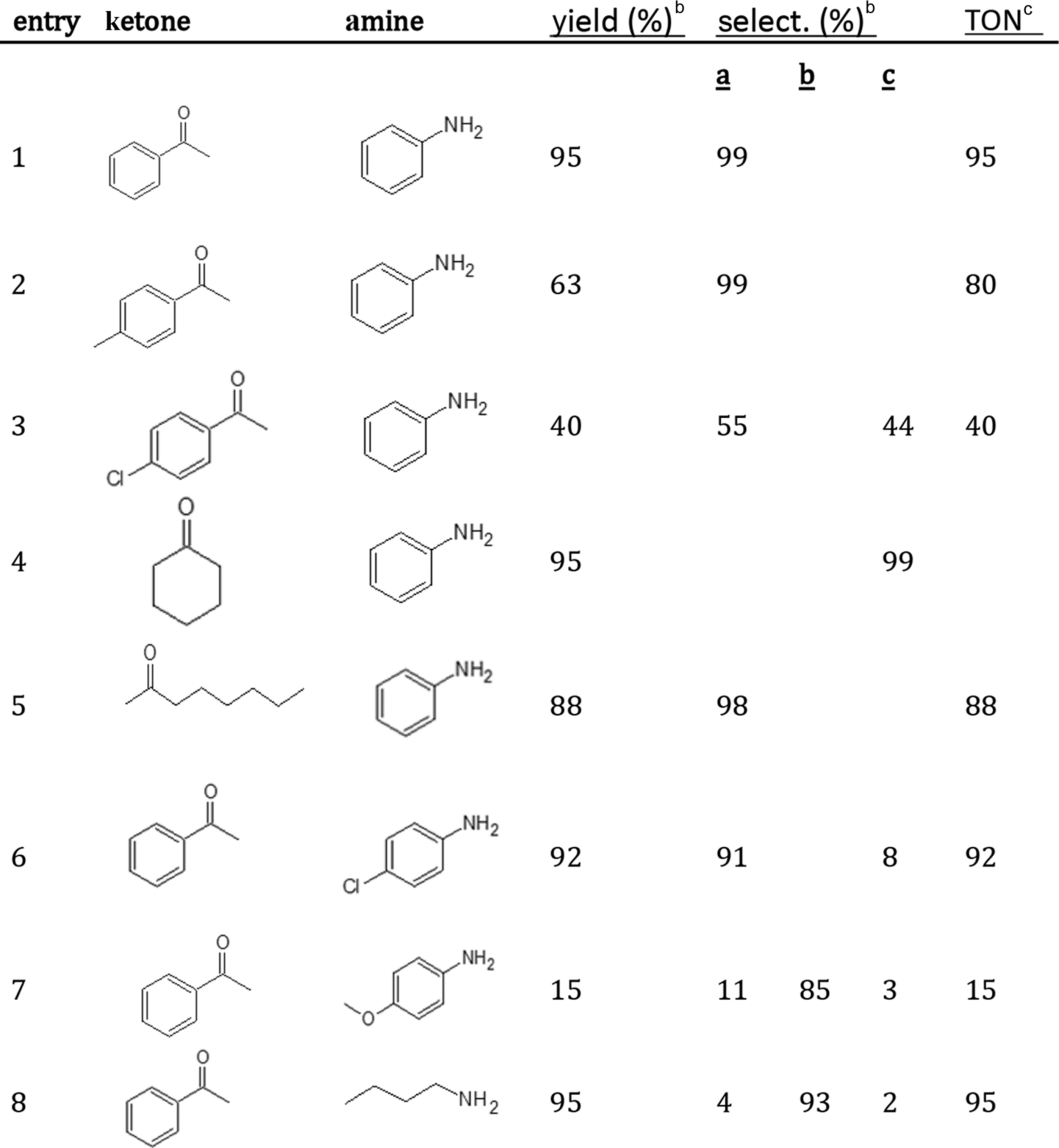
Catalyst Performance in the A^3^ Strecker Reaction Using Various Ketones, Amines, and TMSCN^a^

a Reaction conditions: ketone, amine, and trimethylsilylcianide (1:1:1.1), 1 mol % catalyst is based on **BiPF-4** crystallographic formula **[Bi_14_(μ_3_-O)_9_(μ_4_-O)_2_(μ_3–_OH)_5_(3,5-DSB)_5_(H_2_O)_3_]·7H_2_O, BiPF-4** (4 h), N_2_ atmosphere, room temperature.

b Yield and selectivity calculated by ^1^H-NMR.

c TON = (mmol substrate per mmol catalyst).

The recyclability of the catalyst (**BiPF-4**) was studied, and the catalyst was recovered after centrifugation and washed several times with ethanol and acetone, then dried overnight at 100 °C before each new use. The catalyst was reused at least in nine cycles, showing that even after the ninth run, the selectivity remains unaltered, and the catalytic activity diminishes (yield of 64% in the ninth run) probably due to the loss of material during the recovery of each catalyst (Table S5 and Figure S9). The crystalline structure of materials **BiPF-4** did not suffer any alteration even after the 9th run of reuse, as demonstrated by the PXRD pattern (Figure S10); complete characterization of the Strecker reaction products and spectra for characterized compounds are given in Sections S6 and S7.

Moreover, hot filtration and leaching experiments confirmed that **BiPF-4** is truly a heterogeneous catalyst. The reaction was performed under standard conditions; after 5 min, the catalyst was separated from the reaction media. After 4 h, no progress is observed in the reaction with the liquid fraction, confirming that the presence of the catalyst is necessary in order to selectively complete the organic transformation.

When using **BiPF-4** as the catalyst, the reaction evolves to high yield formation of α-aminonitrile. Assuming that the one-pot Strecker reaction takes place following a mechanism as previously proposed,^37,44^ the formation of the α-aminonitrile requires the activation of both the carbonyl and silyl groups to allow the imine formation, followed by the cleavage of the cyano group and its attack to the imine carbon atom. To the best of our knowledge, this is the first Bi-MOF used as the catalyst in the A^3^ Strecker reaction, and only Bi salts have been used as the catalyst in this type of reaction with aldehydes as carbonyl reactants. **BiPF-4** has demonstrated to be a more efficient heterogeneous catalyst than Bi(NO)_3_^45^ salt because it shows high activity even with the more demanding ketones, using one tenth of the catalyst without any solvent and recyclability proved in at less than 9 cycles.

## Conclusions

In summary, a new robust bismuth MOF has been hydrothermally synthesized under microwave heating in a short reaction time. The new material bears two slightly different SBUs, both composed of seven bismuth atoms with different coordination environments. They are closely disposed, forming extended layers through Bi–O bonds and three-dimensionally packed through the organic linker 3,5-disulfobenzoic acid. Thus, the material is chemically and thermally stable, and exhibits very good activity and selectivity as a heterogeneous catalyst in the multicomponent Strecker reaction carried out in one pot. **BiPF-4** is the first Bi-MOF used as the catalyst in this reaction with ketones, highlighting the potential to access new Lewis acid catalyst with the use of a nontoxic and earth-abundant element such as bismuth.

During the process of evaluation of this study, a 2022 study by T. D. Nguyen et al. appeared on reporting the use of microwave irradiation on the bismuth MOF synthesis.^46^

## Supporting Information Available

Complete synthesis and characterization details, and complete crystallographic information, including CIF file for BiPF-4: [Bi_14_(μ_4_-O)_2_(μ_3_-O)_9_(μ_3–_OH)_5_(3,5-DSB)_5_(H_2_O)_3_]·7H_2_O; complete catalytic details (PDF)

## Accession Codes

CCDC 1963427 contains the supplementary crystallographic data for this paper. These data can be obtained free of charge via www.ccdc.cam.ac.uk/data_request/cif, or by emailing data_request@ccdc.cam.ac.uk, or by contacting The Cambridge Crystallographic Data Centre, 12 Union Road, Cambridge CB2 1EZ, UK; fax: +44 1223 336033.

## References

[ref1] Grape E. S.; Gabriel Flores J.; Hidalgo T.; Martínez-Ahumada E.; Gutierrez-Alejandre A.; Hautier A.; Williams D. R.; O’Keeffe M.; Ohrström L.; Willhammar T.; Horcajada P.; Ibarra I. A.; Ken Inge A. A robust and biocompatible bismuth ellagate MOF synthesized under green ambient conditions. J. Am. Chem. Soc. 2020, 142, 16795–16804. 10.1021/jacs.0c07525. 32894014 PMC7586326

[ref2] Salvador J. A.; Figueiredo S. A.; Pinto R. M.; Silvestre S. M. Bismuth compounds in medicinal chemistry. Future Med. Chem. 2012, 4, 1495–1523. 10.4155/fmc.12.95. 22857536

[ref3] Keogan D. M.; Fagan L. E.; Passos T. M.; Müller-Bunz H.; Quilty B.; Griffith D. M. Synthesis of polymeric bismuth chlorido hydroxamato complexes; X-ray crystal structure and antibacterial activity of a novel Bi(III) salicylhydroxamato complex. Inorgan. Chim. Acta 2017, 460, 134–140. 10.1016/j.ica.2016.09.021.

[ref4] Herdman M. E.; Werrett M. V.; Duffin R. N.; Stephens L. J.; Brammananth R.; Coppel R. L.; Batchelor W.; Andrews P. C. Impact of structural changes in heteroleptic bismuth phosphinates on their antibacterial activity in Bi-nanocellulose composites. Dalton Trans. 2020, 49, 7341–7354. 10.1039/D0DT01226B. 32392274

[ref5] Burke K. J.; Stephens L. J.; Werrett M. V.; Andrews P. C. Bismuth(III) Flavonolates: The Impact of Structural Diversity on Antibacterial Activity, Mammalian Cell Viability and Cellular Uptake. Chem. – Eur. J. 2020, 26, 7657–7671. 10.1002/chem.202000562. 32297355

[ref6] Murafuji T.; Kitagawa K.; Yoshimatsu D.; Kondo K.; Ishiguro K.; Tsunashima R.; Miyakawa I.; Mikata Y. Heterocyclic bismuth carboxylates based on a diphenyl sulfone scaffold: Synthesis and antifungal activity against Saccharomyces cerevisiae. Eur. J. Med. Chem. 2013, 63, 531–535. 10.1016/j.ejmech.2013.02.036. 23535321

[ref7] Babaryk A.; Contreras Almengor O. R.; Cabrero-Antonino M.; Navalón S.; García H.; Horcajada P. A Semiconducting Bi _2_ O _2_ (C _4_ O _4_) Coordination Polymer Showing a Photoelectric Response. Inorg. Chem. 2020, 59, 3406–3416. 10.1021/acs.inorgchem.9b03290. 32077286

[ref8] Singh S. K.; Kumar A.; Gahtori B.; Shruti G. S.; Patnaik S.; Awana V. P. S. Bulk Superconductivity in Bismuth Oxysulfide Bi4O4S3. J. Am. Chem. Soc. 2012, 134, 16504–16507. 10.1021/ja307245a. 23003214

[ref9] Zhao T.; Scholl A.; Zavaliche F.; Lee K.; Barry M.; Doran A.; Cruz M. P.; Chu Y. H.; Ederer C.; Spaldin N. A.; Das R. R.; Kim D. M.; Baek S. H.; Eom C. B.; Ramesh R. Electrical control of antiferromagnetic domains in multiferroic BiFeO3 films at room temperature. Nat. Mater. 2006, 5, 823–829. 10.1038/nmat1731. 16951676

[ref10] Fedorov S. V.; Sedov M. S.; Belousov V. V. Functionally Graded IT-MOFC Electrolytes Based on Highly Conductive δ-Bi _2_ O _3_ −0.2 wt % B _2_ O _3_ Composite with Molten Grain Boundaries. ACS Appl. Energy Mater. 2019, 2, 6860–6865. 10.1021/acsaem.9b01330.

[ref11] Borowska-Centkowska A.; Leszczynska M.; Krok F.; Malys M.; Wrobel W.; Hull S.; Abrahams I. Local structure and conductivity behaviour in Bi 7 WO 13.5. J. Mater. Chem. A 2018, 6, 5407–5418. 10.1039/C7TA09225C.

[ref12] Gu B.; Peron D. V.; Barrios A. J.; Virginie M.; La Fontaine C.; Briois V.; Vorokhta M.; Šmíd B.; Moldovan S.; Koneti S.; Gambu T. G.; Saeys M.; Ordomsky V. V.; Khodakov A. Y. Bismuth mobile promoter and cobalt-bismuth nanoparticles in carbon nanotube supported Fischer-Tropsch catalysts with enhanced stability. J. Catal. 2021, 401, 102–114. 10.1016/j.jcat.2021.07.011.

[ref13] Thompson M. C.; Ramsay J.; Weber J. M. Solvent-Driven Reductive Activation of CO 2 by Bismuth: Switching from Metalloformate Complexes to Oxalate Products. Angew. Chem., Int. Ed. 2016, 55, 15171–15174. 10.1002/anie.201607445. 27730755

[ref14] Planas O.; Wang F.; Leutzsch M.; Cornella J. Fluorination of arylboronic esters enabled by bismuth redox catalysis. Science 2020, 367, 313–317. 10.1126/science.aaz2258. 31949081

[ref15] Brazdil J. F.; Toft M. A.; Lin S. S.-Y.; McKenna S. T.; Zajac G.; Kaduk J. A.; Golab J. T. Characterization of bismuth–cerium-molybdate selective propylene ammoxidation catalysts. Appl. Catal., A 2015, 495, 115–123. 10.1016/j.apcata.2015.02.012.

[ref16] Roy K.; Artiglia L.; Xiao Y.; Varma A.; van Bokhoven J. A. Role of Bismuth in the Stability of Pt–Bi Bimetallic Catalyst for Methane Mediated Deoxygenation of Guaiacol, an APXPS Study. ACS Catal. 2019, 9, 3694–3699. 10.1021/acscatal.8b04699.

[ref17] Poudel B.; Hao Q.; Ma Y.; Lan Y.; Minnich A.; Yu B.; Yan X.; Wang D.; Muto A.; Vashaee D.; Chen X.; Liu J.; Dresselhaus M. S.; Chen G.; Ren Z. High-Thermoelectric Performance of Nanostructured Bismuth Antimony Telluride Bulk Alloys. Science 2008, 320, 634–638. 10.1126/science.1156446. 18356488

[ref18] Tran D. T.; Chu D.; Oliver A. G.; Oliver S. R. J. A 3-D bismuth–organic framework containing 1-D cationic inorganic [Bi_2_O_2_]^2+^ chains. Inorg. Chem. Commun. 2009, 12, 1081–1084. 10.1016/j.inoche.2009.08.030.

[ref19] Wang G.; Sun Q.; Liu Y.; Huang B.; Dai Y.; Zhang X.; Qin X. A Bismuth-Based Metal-Organic Framework as an Efficient Visible-Light-Driven Photocatalyst. Chem. – Eur. J. 2015, 21, 2364–2367. 10.1002/chem.201405047. 25487284

[ref20] Köppen M.; Beyer O.; Wuttke S.; Lüning U.; Stock N. Synthesis, functionalisation and post-synthetic modification of bismuth metal-organic frameworks. Dalton Trans. 2017, 46, 8658–8663. 10.1039/C7DT01744H. 28650040

[ref21] Vilela S. M. F.; Babaryk A. A.; Jaballi R.; Salles F.; Mosquera M. E. G.; Elaoud Z.; Van Cleuvenbergen S.; Verbiest T.; Horcajada P. A Nonlinear Optically Active Bismuth-Camphorate Coordination Polymer. Eur. J. Inorg. Chem. 2018, 2018, 2437–2443. 10.1002/ejic.201800197.

[ref22] Sun Y. Q.; Ge S. Z.; Liu Q.; Zhong J. C.; Chen Y. P. A novel 3-D chiral bismuth-organic framework with mixed carboxylate, pyridine and thiolate donors exhibiting a semiconductive property. CrystEngComm 2013, 15, 10188–10192. 10.1039/c3ce41696h.

[ref23] Feyand M.; Mugnaioli E.; Vermoortele F.; Bueken B.; Dieterich J. M.; Reimer T.; Kolb U.; De Vos D.; Stock N. Automated diffraction tomography for the structure elucidation of twinned, sub-micrometer crystals of a highly porous, catalytically active bismuth metal-organic framework. Angew. Chem., Int. Ed. 2012, 51, 10373–10376. 10.1002/anie.201204963. 22976879

[ref24] Wang Q.-X.; Li G. Bi(iii) MOFs: syntheses, structures and applications. Inorg. Chem. Front. 2021, 8, 572–589. 10.1039/D0QI01055C.

[ref25] Feyand M.; Köppen M.; Friedrichs G.; Stock N. Bismuth tri- and tetraarylcarboxylates: Crystal structures, in situ X-ray diffraction, intermediates and luminescence. Chem. – Eur. J. 2013, 19, 12537–12546. 10.1002/chem.201301139. 23897702

[ref26] Wang Z.; Zeng Z.; Wang H.; Zeng G.; Xu P.; Xiao R.; Huang D.; Chen S.; He Y.; Zhou C.; Cheng M.; Qin H. Bismuth-based metal–organic frameworks and their derivatives: Opportunities and challenges. Coord. Chem. Rev. 2021, 439, 213902 10.1016/j.ccr.2021.213902.

[ref27] Gomez G. E.; D’vries R. F.; Lionello D. F.; Aguirre-Díaz L. M.; Spinosa M.; Costa C. S.; Fuertes M. C.; Pizarro R. A.; Kaczmarek A. M.; Ellena J.; Rozes L.; Iglesias M.; Van Deun R.; Sanchez C.; Monge M. A.; Soler-Illia G. J. A. A. Exploring physical and chemical properties in new multifunctional indium-, bismuth-, and zinc-based 1D and 2D coordination polymers. Dalton Trans. 2018, 47, 1808–1818. 10.1039/C7DT04287F. 29322149

[ref28] Inge A. K.; Köppen M.; Su J.; Feyand M.; Xu H.; Zou X.; O’Keeffe M.; Stock N.; O’Keeffe M.; Stock N. Unprecedented Topological Complexity in a Metal-Organic Framework Constructed from Simple Building Units. J. Am. Chem. Soc. 2016, 138, 1970–1976. 10.1021/jacs.5b12484. 26790573

[ref29] Savage M.; Yang S.; Suyetin M.; Bichoutskaia E.; Lewis W.; Blake A. J.; Barnett S. A.; Schröder M. A Novel Bismuth-Based Metal-Organic Framework for High Volumetric Methane and Carbon Dioxide Adsorption. Chem. – Eur. J. 2014, 20, 8024–8029. 10.1002/chem.201304799. 24827914

[ref30] García-Sánchez A.; Gomez-Mendoza M.; Barawi M.; Villar-Garcia I. J.; Liras M.; Gándara F.; de la Peña O’Shea V. A. Fundamental Insights into Photoelectrocatalytic Hydrogen Production with a Hole-Transport Bismuth Metal–Organic Framework. J. Am. Chem. Soc. 2020, 142, 318–326. 10.1021/jacs.9b10261. 31809033

[ref31] Albat M.; Inge A. K.; Stock N. Synthesis and crystal structure of three new bismuth(III) arylsulfonatocarboxylates. Z. Kristallogr. - Cryst. Mater. 2017, 232, 245–253. 10.1515/zkri-2016-1980.

[ref32] Albat M.; Stock N. Multiparameter High-Throughput and in Situ X-ray Diffraction Study of Six New Bismuth Sulfonatocarboxylates: Discovery, Phase Transformation, and Reaction Trends. Inorg. Chem. 2018, 57, 10352–10363. 10.1021/acs.inorgchem.8b01563. 30070474

[ref33] Senevirathna D. C.; Blair V. L.; Werrett M. V.; Andrews P. C. Polynuclear Bismuth Oxido Sulfonato Clusters, Polymers, and Ion Pairs from Bi_2_O_3_ under Mild Conditions. Inorg. Chem. 2016, 55, 11426–11433. 10.1021/acs.inorgchem.6b01937. 27739672

[ref34] D’Vries R. F.; de la Peña-O’Shea V. A.; Snejko N.; Iglesias M.; Gutiérrez-Puebla E.; Monge M. A. H_3_O_2_ Bridging Ligand in a Metal–Organic Framework. Insight into the Aqua-Hydroxo↔Hydroxyl Equilibrium: A Combined Experimental and Theoretical Study. J. Am. Chem. Soc. 2013, 135, 5782–5792. 10.1021/ja4005046. 23510527

[ref35] D’Vries R. F.; Álvarez-García S.; Snejko N.; Bausá L. E.; Gutiérrez-Puebla E.; de Andrés A.; Monge M. Á. Multimetal rare earth MOFs for lighting and thermometry: tailoring color and optimal temperature range through enhanced disulfobenzoic triplet phosphorescence. J. Mater. Chem. C 2013, 1, 6316 10.1039/c3tc30858h.

[ref36] D’Vries R. F.; de la Peña-O’Shea V. A.; Benito Hernández Á.; Snejko N.; Gutiérrez-Puebla E.; Monge M. A. Enhancing Metal–Organic Framework Net Robustness by Successive Linker Coordination Increase: From a Hydrogen-Bonded Two-Dimensional Supramolecular Net to a Covalent One Keeping the Topology. Cryst. Growth Des. 2014, 14, 5227–5233. 10.1021/cg501028f.

[ref37] aAguirre-Díaz L. M.; Gándara F.; Iglesias M.; Snejko N.; Gutiérrez-Puebla E.; Monge M. Á. Tunable Catalytic Activity of Solid Solution Metal–Organic Frameworks in One-Pot Multicomponent Reactions. J. Am. Chem. Soc. 2015, 137, 6132–6135. 10.1021/jacs.5b02313. 25843316

[ref38] aStrecker A. Ueber die kiinstliche Bildung der Milchsaure undeinen neuen, dem Glycocoll homologen Korper. Justus Liebigs Ann. Chem. 1850, 75, 27 10.1002/jlac.18500750103.

[ref39] Coxall R. A.; Harris S. G.; Henderson D. K.; Parsons S.; Tasker P. A.; Winpenny R. E. P. Inter-ligand reactions: in situ formation of new polydentate ligands. J. Chem. Soc., Dalton Trans. 2000, 2349–2356. 10.1039/b001404o.

[ref40] Andrews P. C.; Busse M.; Junk P. C.; Forsyth C. M.; Peiris R. Sulfonato-encapsulated bismuth(III) oxido-clusters from Bi_2_O_3_ in water under mild conditions. Chem. Commun. 2012, 48, 7583–7585. 10.1039/c2cc33495j. 22735330

[ref41] Gándara F.; Gutiérrez-Puebla E.; Iglesias M.; Snejko N.; Monge M. Á. Isolated Hexanuclear Hydroxo Lanthanide Secondary Building Units in a Rare-Earth Polymeric Framework Based on p -Sulfonatocalix[4]arene. Cryst. Growth Des. 2010, 10, 128–134. 10.1021/cg900704x.

[ref42] Luebke R.; Belmabkhout Y.; Weseliński Ł. J.; Cairns A. J.; Alkordi M.; Norton G.; Wojtas Ł.; Adil K.; Eddaoudi M. Versatile rare earth hexanuclear clusters for the design and synthesis of highly-connected ftw -MOFs. Chem. Sci. 2015, 6, 4095–4102. 10.1039/C5SC00614G. 29218176 PMC5707464

[ref43] Köppen M.; Dhakshinamoorthy A.; Inge A. K.; Cheung O.; Ångström J.; Mayer P.; Stock N. Synthesis, Transformation, Catalysis, and Gas Sorption Investigations on the Bismuth Metal-Organic Framework CAU-17. Eur. J. Inorg. Chem. 2018, 2018, 3496–3503. 10.1002/ejic.201800321.

[ref44] Aguirre-Díaz L. M.; Iglesias M.; Snejko N.; Gutiérrez-Puebla E.; Monge M. Á. Synchronizing Substrate Activation Rates in Multicomponent Reactions with Metal-Organic Framework Catalysts. Chem. – Eur. J. 2016, 22, 6654–6665. 10.1002/chem.201504576. 27010759

[ref45] Mansoor S. S.; Aswin K.; Logaiya K.; Sudhan S. P. N. An efficient one-pot three-component synthesis of a-amino nitriles via Strecker reaction catalyzed by bismuth(III) nitrate. J. Saudi Chem. Soc. 2016, 20, S202–S210. 10.1016/j.jscs.2012.10.009.

[ref46] Nguyen V. H.; Phamc A. L. H.; Nguyend V.-H.; Leee T.; Nguyen T. D. Facile synthesis of bismuth(III) based metal-organic framework with difference ligands using microwave irradiation method. Chem. Eng. Res. Des. 2022, 177, 321–330. 10.1016/j.cherd.2021.10.043.

