# Supplementary material for: Building a Green, Robust, and Efficient Bi-MOF Heterogeneous Catalyst for the Strecker Reaction of Ketones
Source: Inorg Chem. 2022 May 5;61(19):7523–9. doi: 10.1021/acs.inorgchem.2c00628 (PMC9115759; doi:10.1021/acs.inorgchem.2c00628)
Supplement: Supplementary file 1 — ic2c00628_si_001.pdf [file ic2c00628_si_001.pdf]

Supporting material

Building a Green and Robust Efficient Bi-MOF Heterogeneous Catalyst for the Strecker Reaction of Ketones

Eloy P. Gómez-Oliveira, Nayara Méndez, Marta Iglesias, Enrique Gutiérrez-Puebla, Lina M. Aguirre-Díaz, and M. Ángeles Monge **

† Materials Science Institue of Madrid – Spanish National Research Council (ICMM-CSIC), Sor Juana Inés de la Cruz 3, Cantoblanco 28049, Madrid, Spain.

Keywords: Metal-organic frameworks, Bismuth MOF, One pot heterogeneous catalysis. Strecker reaction.

Corresponding Authors
*E-mail: amonge@icmm.csic.es.
*E-mail: aguirrediaz.lina@gmail.com

S1. Experimental procedure

- General
- Synthesis of BiPF-4 and characterization
- Catalytic activity experiments procedure

S2. Single crystal X-ray diffraction and structure of BiPF-4
S3. Catalytic activity experiments background
S4. Catalytic activity data
S5. Recycle reactions
S6. Characterization of the Strecker 3C reaction products

- Characterization of 3C Strecker Reaction ketone and amine scope

S7. Spectra for characterized compounds
S8. References

- **S1. EXPERIMENTAL PROCEDURE**

1.1. General

All reagents and solvents employed were commercially available and used as received without further purification. Bismuth Nitrate (III) pentahydrate, $Bi(NO_3)_3 \cdot 5 H_2O$ (from Fluka); 3,5-disulfobenzoic acid, disodium salt (3,5-DSB, purchased from Aldrich Chemical Company); Dichloromethane (DCM, obtained from Labkem); Ethanol Absolute (EtOH, obtained from Scharlau); Acetone, 99,5% (purchased from Labkem). Solvothermal synthesis mediated by microwave is carried out in a CEM Discover SP microwave oven, performing the synthesis experiments using a dynamic method with variable power (max. 200 W) and fixed temperature (180 ºC), pre-stirring (1 min), stirring during the synthesis, and cooling ramp of 50 ºC/min until to 60 ºC. NMR spectra were recorded in a Bruker Advance 300-II equipment using $CDCl_3$ as solvent. IR spectra were recorded from KBr pellets in the range 4000-400 cm^{-1} on a Bruker Vertex 70V spectrophotometer, with a resolution of 2 cm^{-1}. Thermogravimetric and differential thermal analysis (TGA-DTA) was performed using a SDT Q600 from TA Instruments equipment in a temperature range between 30 and 800 ºC in N_2 or air (100 mL/min flow) atmosphere and heating rate of 10ºC/min. A CNHS PERKIN ELMER 2400 analyzer was employed for the elemental analysis. Powder X-ray diffraction (PXRD) patterns were measured with a Bruker D8 diffractometer with a copper source operated at 1600 W, with step size = 0.02º and exposure time = 0.5 s/step. PXRD measurements were used to check the purity of the obtained microcrystalline products by a comparison of the experimental results with the simulated patterns obtained from single-crystal X-ray diffraction data.

1.2 Synthesis of BiPF-4 and Characterization

$\{[Bi_{14}(\mu_4\text{-}O)_2(\mu_3\text{-}O)_9(3,5\text{-}DSB)_5(OH)_5(H_2O)_3]\cdot 7H_2O\}$: In a microwave tube, 100 mg of $Bi(NO_3)_3\cdot 5H_2O$ (0,20 mmol) and 66 mg of 3,5-disulfobenzoic acid, disodium salt (3,5-DSB; 0,20 mmol) were added. We also add 4 mL of distilled water to the microwave tube, and we introduce it in the microwave at 180 ºC for one hour. Pure, white, sheet-like crystals were obtained after being centrifuged and washed with water, ethanol and acetone. Powder X-ray diffraction supported the purity of the sample. Yield: 51 mg, 74%. Elemental analysis, found (calculated): C, 8.83 (8.75); H, 1.02 (0.97). IR (KBr, cm^{-1}): 3434 ν(O-H), 1602 ν(C=O), 1536 ν(C-O), 1363 ν(C-S), 1201, 1112, 1033, 639 and 610 ν(C-C) aromatic, 784 ν(C-C), 519 and 431 ν(Bi-O).

Formula **{**$[Bi_{14}(\mu_4\text{-}O)_2(\mu_3\text{-}O)_9(3,5\text{-}DSB)_5(OH)_5(H_2O)_3]\cdot L\}$ **BiPF-4,** CCDC number 1963427. **Formula weight:** 4641.79 + L (L = ethanol, water)

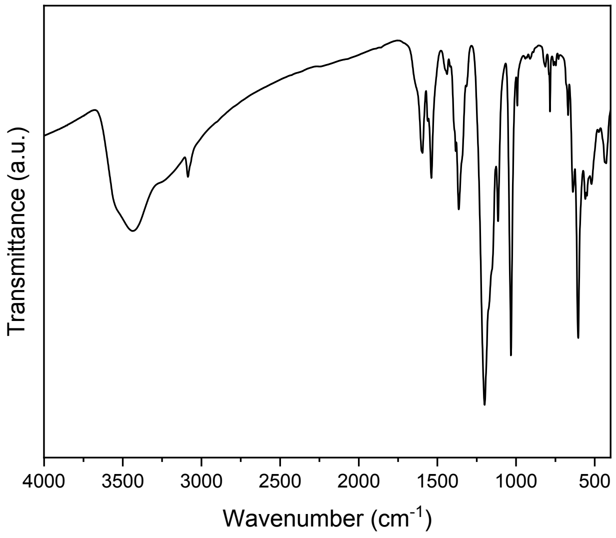

Figure S1. Infrared transmittance spectra of **BiPF-4** MOF (KBr; cm^{-1}).

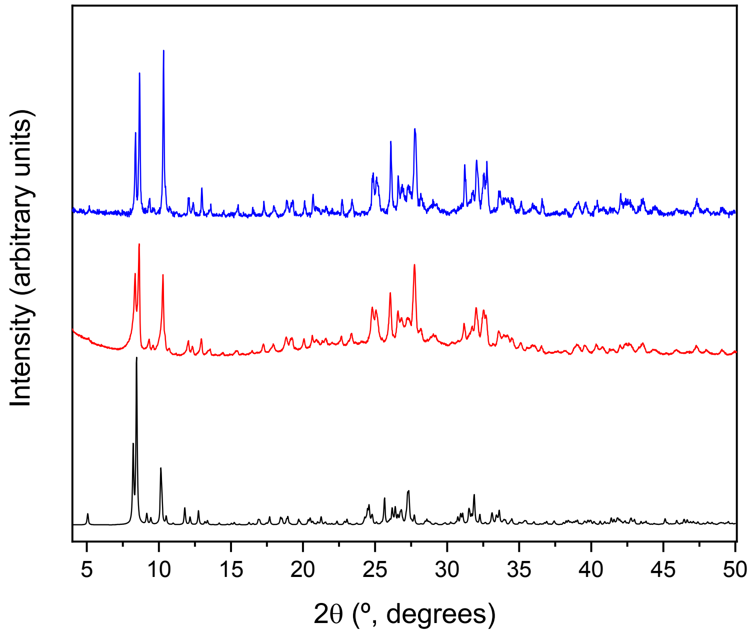

Figure S2. X-ray powder spectra, showing the purity of de sample before and after activation. Bottom up, **BiPF-4** as-activated (blue), synthesized (red) and simulated from single crystal data (black).

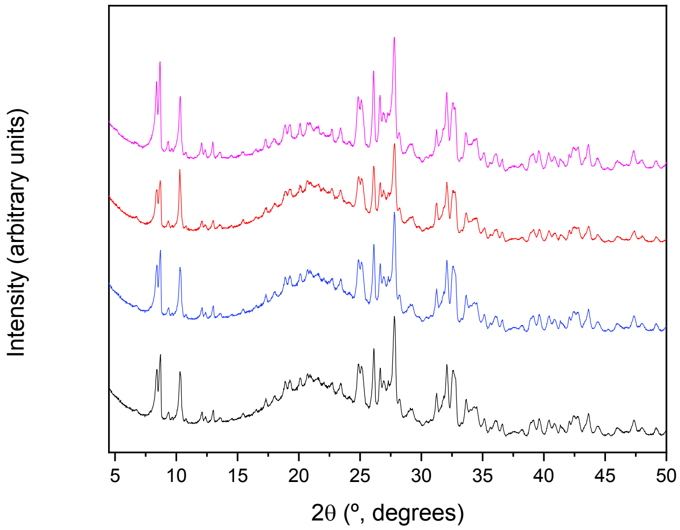

Figure S3. X-ray powder spectra**.** From bottom to top, **BiPF-4** activated (black), activated after soaking 4 hours in distilled water (blue), in a solution at pH=2 (red) and in a solution at pH=9 (pink).

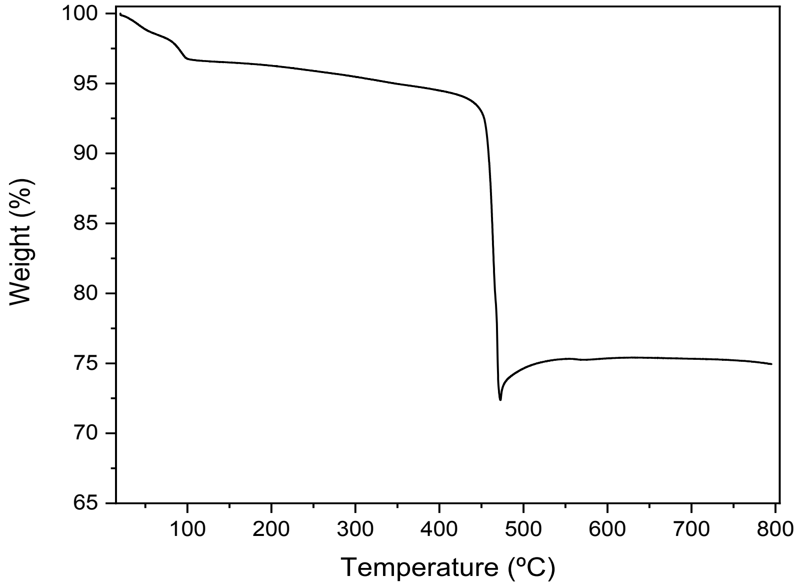

Figure S4. TG-DTA curve for as-synthesized **BiPF-4** in air. Pores are filled with acetone and water molecules, which evacuates from 60 to 110ºC, corresponding to a loss of 4 % of th0e total weight. The structure finally collapses when temperature reaches 470ºC. The final residue is a mixture of monoclinic Bi_2O_3 and $Bi_3S_2O_{2.5}$.

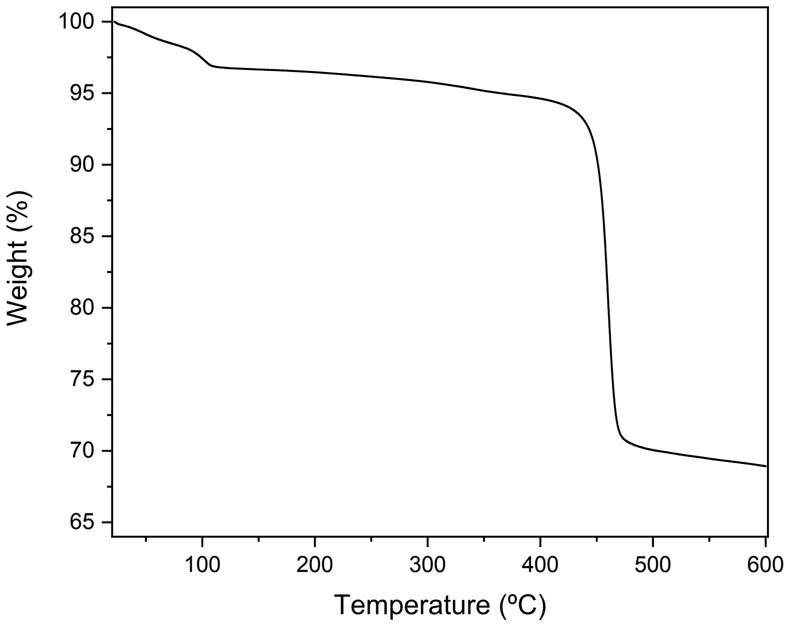

Figure S5. TG-DTA curve for as-synthesized **BiPF-4** in N_2. Pores are filled with acetone and water molecules, which evacuates from 60 to 110ºC, corresponding to a loss of 4 % of the total weight. The structure finally collapses when temperature reaches 470ºC. The final residue is metallic bismuth. PDF Card ICDD 00-044-1246.

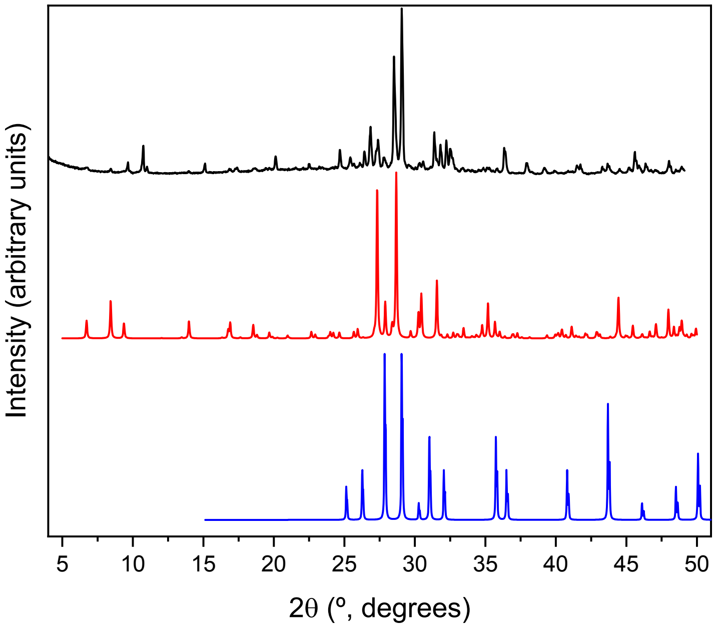

Figure S6. Powder X-ray diffraction of residues after thermogravimetric analysis (black), fitting according to a mixture of two products, $Bi_{14}(SO_4)_5O_{16}$ PXRD PDF Card LPF 04-022-0198 (red), and $Bi_8(SO_4)_3O_9$ PDXR PDF Card ICDD pattern 00-021-0820 (blue).

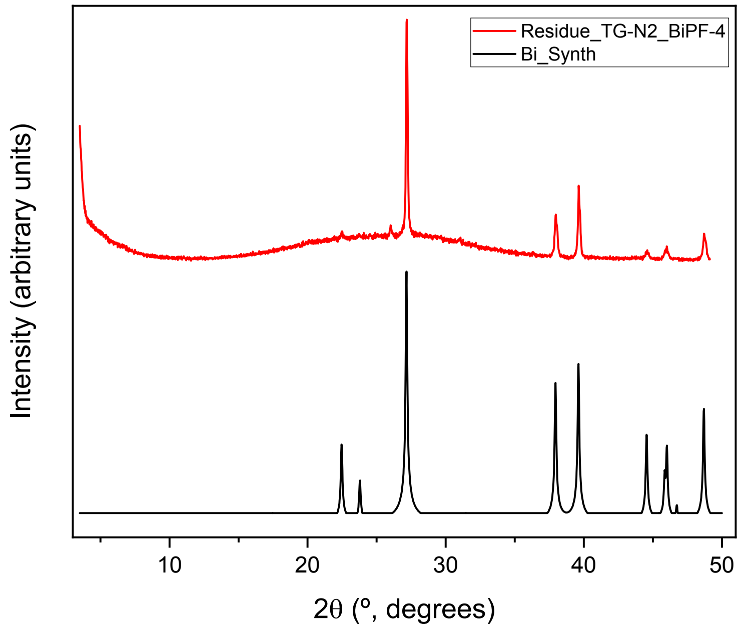

Figure S7. Powder X-ray diffraction of residues after thermogravimetric analysis (red), fitting according to metallic Bi. PXRD PDF Card ICDD 00-044-1246 (black).

1.3 Catalytic activity experiments procedure

Catalyst was activated under vacuum for a period of 18 hours after soaking three days in the corresponding solvent (water, ethanol and acetone), which was changed four times per day. Then, the mixture of ketone, amine and trimethylsilyl cyanide (TMSCN) was added to a Schlenk tube (1.89 mmol of ketone, 1.89 mmol of amine and 1.98 mmol of TMSCN), where the MOF has been previously introduced. The mixture (in solvent-free conditions) was stirred (300 rpm) at 25ºC between 2.5 and 24 h depending on the amount of catalyst, under N_2 atmosphere. For comparative purpose, the same standard reaction conditions were used for all tested substrates, and therefore the reaction parameters were not further optimized for each one of them. The completion of the reaction was monitored by ^{1}H-NMR. When reaction was completed and samples were taken, the content of the Schlenk tube was mixed with DCM in order to dissolve the Strecker α-aminonitrile and recover the catalyst by centrifugation. X-ray diffraction patterns were obtained before and after each experiment to ensure crystallinity, purity and recyclability of the MOF.

Catalyst was reused at least in 9 cycles. In each case, after recovery, BiPF-4 was washed with ethanol and acetone and dried overnight at 100 ºC following the same procedure described above, without significant loss of activity (yield of 64% in the nineth run). The leaching test was performed as follows. The mixture remained 5 minutes within the conditions previously described. After that, part of the liquid was separated and transferred to an empty Schlenk. After 4 hours, no progress is observed in the reaction. After 24 hours, different products were found in the mixture, mainly composed of unreacted acetophenone (20%) and aniline (22%), a small amount of aminonitrile (15%), and other undetermined products (scheme 1 shows the main possible products). This probed that the presence of the catalyst is necessary in order to selectively complete the organic transformation.

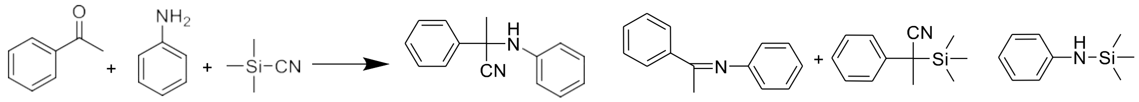

Scheme S1. Strecker reaction and possible by-products: α-aminonitrile, imine, cyanohydrin and silylamine

- **S2. X-RAY STRUCTURE DETERMINATION**

2.1 Main crystallographic data for BiPF-4

Table S1. summarizes the main crystal and refinement data for BiPF-4. Crystals were selected under a polarizing optical microscope for a singlecrystal X-ray diffraction experiment. Single-crystal X-ray data were obtained in a Bruker four circle kappa-diffractometer equipped with a Cu INCOATED microsource, operated at 30 W power (45kV, 0.60mA) to generate Cu Kα radiation (λ = 1.54178 Å), and a Bruker VANTEC 500 area detector (microgap technology). Diffraction data were collected exploring over a hemisphere of the reciprocal space in a combination of φ and ω scans to reach a resolution of 0.85 Å, using a Bruker APEX2[1] software suite (each exposure, depending on ω, was of 56, 90 or 140 s covering 1° in ω or φ). Unit cell dimensions were determined for least-squares fit of reflections with I>4σ. The structures were solved by intrinsic phase methods[2]. The hydrogen atoms were fixed at their calculated positions using distances and angle constraints. All calculations were performed using APEX2 software for data collection and OLEX2-1.2[3] and SHELXTL[4] to resolve and refine the structure.

Table S1. Main crystallographic data for **BiPF-4** compound. Data recovered from BiPF-4 crystal after evacuation.

**Identification code**	**BiPF-4**
**Empirical formula**	$C_{35}H_{15}Bi_{14}O_{60}S_{10}$
**Formula weight**	4641.79
**Temperature/K**	296.15
**Crystal system**	triclinic
**Space group**	*P1*
**a/Å**	10.915(14)
**b/Å**	11.108(12)
**c/Å**	17.648(18)
**α/°**	90.59(3)
**β/°**	98.45(4)
**γ/°**	104.26(4)
**Volume/Å^3**	2049(4)
**Z**	1
**ρ_{calc}g/cm^3**	3.762
**μ/mm^{-1}**	61.073
**F(000)**	2027.0
**Crystal size/mm^3**	0.05 × 0.04 × 0.05
**Radiation**	CuKα (λ = 1.54184)
**2Θ range for data collection/°**	5.068 to 125.076
**Index ranges**	-12 ≤ h ≤ 12, -12 ≤ k ≤ 12, -20 ≤ l ≤ 20
**Reflections collected**	39987
**Independent reflections**	12288 [R_{int} = 0.0948, R_{sigma} = 0.1125]
**Data/restraints/parameters**	12288/3/509
**Goodness-of-fit on F^2**	0.950
**Final R indexes [I>=2σ (I)]**	R_1 = 0.0664, wR_2 = 0.1711
**Final R indexes [all data]**	R_1 = 0.0882, wR_2 = 0.1969
**Largest diff. peak/hole / e Å^{-3}**	2.50/-2.95
**Flack parameter**	0.44(4)

- **S3. CATALYTIC ACTIVITY EXPERIMENTS BACKGROUND**

Table S2. Catalytic activity of several homogeneous and heterogeneous catalysts used in the acetophenone mediated 3C-Strecker reaction.

Catalyst	Type	Reaction conditions	Time and yield	Reference
o-Benzenedisulfonimide	homogeneous	2-5 mol%, 20-25ºC	5 min-6 h; 93-95 %	Ghigo *et al.*[6]
Sulfonic acid based nanoreactors	heterogeneous	5 mol%, r.t., solvent-free	55 min; 98%	Karimi *et al.*[7]
Nafion-Fe	heterogeneous	100 mg, 80ºC, DCM	6 h; 62 %	Mathew *et al.*[8]
$Ga(OTf)_3$	heterogeneous	5 mol%, r.t., DCM	5 h; 98 %	Olah *et al.*[9]
BINOL-derives phosphoric acids	homogeneous	10 mol%, 40ºC, toluene	24-48 h; 98 %	Jun-An Ma *et al.*[10]
$InBr_3$	homogeneous	10 mol%, up to 80ºC, toluene	27 h; 96 %	Sakai *et al.*[11]

Table S3. Catalytic activity of different *p*-MOFs in the A^3-Strecker reaction with acetophenone, aniline and trimethylsilyl cyanide.

Catalyst	Type	Reaction conditions	Time and yield	Reference
In-MOF 1	heterogeneous	50 mol%, r.t., CDCl3 (2C)	96 h; 46.5 %	Jifu Zheng *et al.*[12]
In-MOF 2	heterogeneous	50 mol%, r.t., CDCl3 (2C)	96 h; 99.6 %	Jifu Zheng *et al.*[12]
InGaPF-1	heterogeneous	1 mol%, r.t., Solvent-free	0.42 h; 50 %	Monge *et al.*[13]
InGaPF-2	heterogeneous	1 mol%, r.t., Solvent-free	0.75 h; 67 %	Monge *et al.*[13]
InGaPF-3	heterogeneous	1 mol%, r.t., Solvent-free	2 h; 80 %	Monge *et al.*[13]
InPF-11α	heterogeneous	2.5 mol%, 80ºC, Solvent-free	4 h; 65 %	Monge *et al.*[13]
InPF-11β	heterogeneous	1 mol%, r.t., Solvent-free	0.67 h; 67 %	Monge *et al.*[13]
GaPF-1	heterogeneous	1 mol%, r.t., Solvent-free	0.63 h; 67 %	Monge *et al.*[13]
AlPF-1	heterogeneous	1 mol, r.t., Solvent-free	8 h; 75 %	Monge *et al.*[13]
$Bi(NO)_3$*	heterogeneous	10 mol% CH_3CN	1h; 94%	S. Sheik Mansoor et all[14]

* *Proved only with benzaldehyde not with ketones*

- **S4. CATALYTIC ACTIVITY DATA**

Table S4. Screening of catalyst loadings and scaled up in the Strecker one-pot three component reaction using acetophenone, aniline and TMSCN (1:1:1.1) [a].

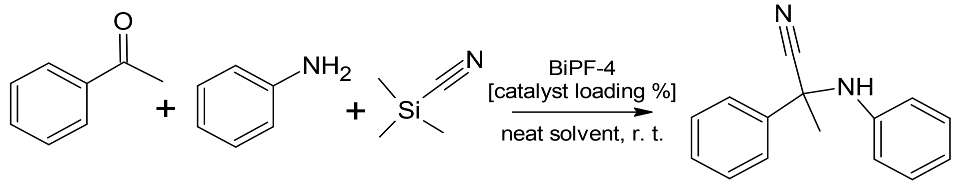

Entry	Catalyst loading (mol%)	Time (h)	Yield[b] (%)	TON[c]
1	0.25	4	20	80
2	0.5	4	40	80
3	1	4	95	95
4	2	2.8	99	49
5	1*	4	86	86
6	1**	4	74	74
7	–	6	4	–
8	1***	4	12	12

[a] *Reaction conditions*: acetophenone, amine, TMSCN (1:1:1.1), mol% catalyst based on $\{[Bi_{14}(\mu_4\text{-}O)_2(\mu_3\text{-}O)_9(OH)_5(3,5\text{-}DSB)_5(H_2O)_3]\cdot 7H_2O\}$, N_2 atmosphere, 25 °C; catalyst washed with dichloromethane. [b] Yield by ^1H NMR. [c] TON = (mmol substrate/ mmol catalyst). * Scaled up x3. ** Scaled up x5. *** Leaching test; 5 min with catalyst, centrifugate to separate the reactants from the catalyst, and allow to react without catalyst.

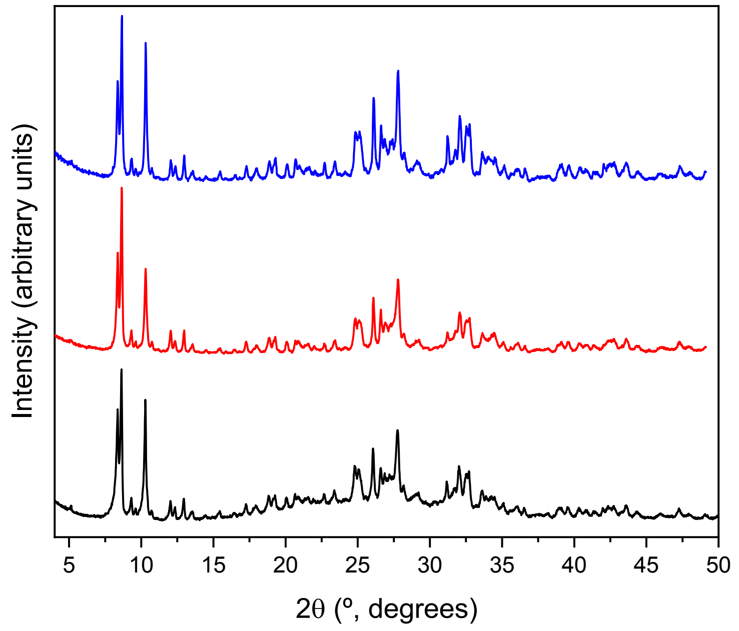

Figure S8. Bottom up, **BiPF-4** as-synthesized (blue), activated (red) and after standard catalytic reaction (black).

- **S5. RECYCLE REACTIONS**

Table S5. Strecker one-pot three component reaction using acetophenone, aniline and TMSCN (1:1:1.1) up to 9 cycles.

**Run**[a]	**Yield (%)**[b]	**TON**[c]
1	95	95
2	96	96
3	89	89
4	82	82
5	77	77
6	73	73
7	69	69
8	66	66
9	63	63

[a] *Reaction conditions*: N2 atmosphere, without solvent, at room temperature (25ºC), in 4 hours, with a catalyst loading of 1 mol%. [b] Yield calculated by ^{1}H NMR from reaction crude. [C] TON = (mol substrate/ mol catalyst).

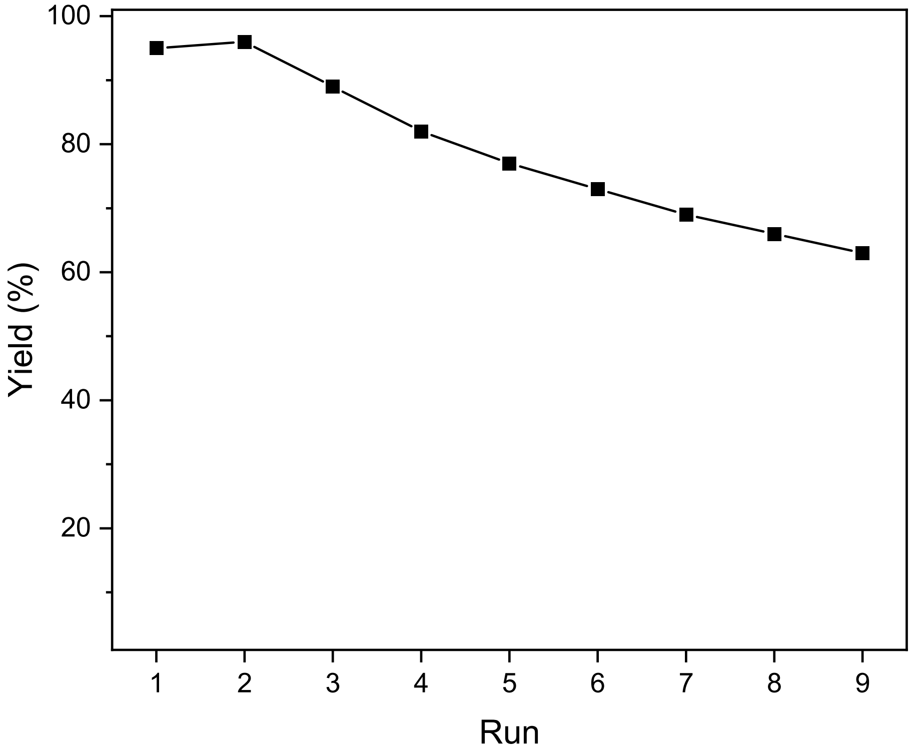

Figure S9. Graph with recycle run yields obtained, from the first to the ninth.

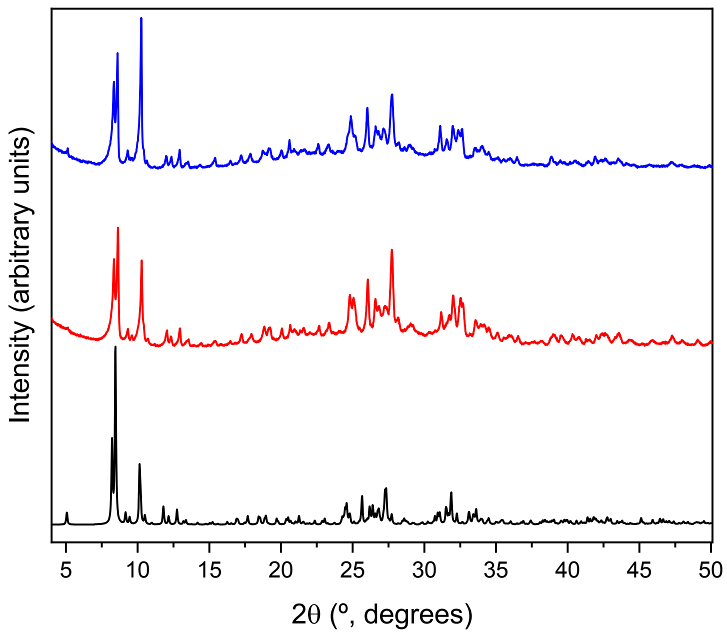

Figure S10. Simulated profile from single-crystal diffraction structure data (black), experimental profile from powder X-ray diffraction (red) and after the nineth run in catalytic activity experiments (blue).

- **S6. CHARACTERIZATION OF THE STRECKER 3C REACTION PRODUCT**

Characterization of 3C Strecker Reaction ketone and amine scope from Table1

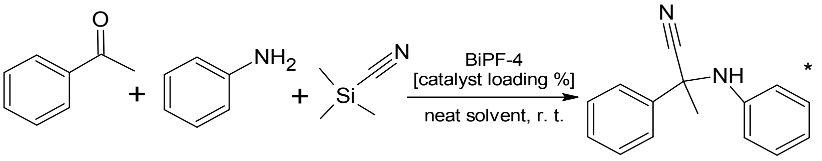

Entry 1 *2-Phenyl-2-(phenylamino)propanenitrile
White solid: ^{1}H NMR (300 MHz, $CDCl_3$): δ = 1.94 (s, 3H), 4.33 (brs, 1H, NH), 6.55 (d, 2H), 6.81 (t, 1H), 7.13 (t, 2H), 7.40 (m, 3H), 7.64 (m, 2H); which corresponds with the spectrum reported for compound with CAS No. 17424-68-9. This compound isknown.[6]

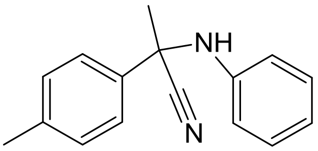

Entry 2. 2-(phenylamino)-2-(*p*-tolyl)propanenitrile [6]
CAS Registry Number: 17424-69-0.
^{1}H NMR (300 MHz; $CDCl_3$): δ = 1.95 (s, 3H), 2.38 (s, 3H), 4.27 (brs, 1H, NH), 6.58 (d, 2H), 6.83 (t, 1H), 7.11-7.17 (m, 2H), 7.23 (d, 2H), 7.52 (d, 2H)

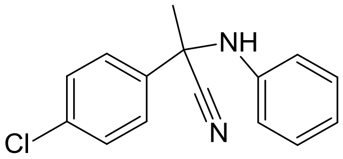

Entry 3. 2-(4-chlorophenyl)-2-(phenylamino)propanenitrile [14]
CAS Registry Number: 17424-70-3.
^{1}H NMR (300 MHz; $CDCl_3$): δ = 1.94 (s, 3H), 4.28 (brs, 1H, NH), 6.52-6.55 (d, 2H), 6.81-6.86 (t, 1H), 7.12-7.17 (m, 2H), 7.37-7.40 (d, 2H), 7.56-7.59 (d, 2H)

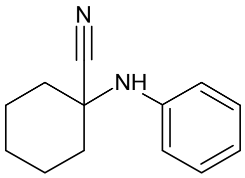

Entry 4. 1-(phenylamino)cyclohexanecarbonitrile [16]
CAS Registry Number: 64269-06-3.
^{1}H NMR (300 MHz; $CDCl_3$): δ = 1.26-1.37 (m, 1H), 1.61-1.82 (m, 7H), 2.32-2.41 (m, 2H), 3.26 (brs, 1H, NH), 6.93 (m, 3H), 7.25 (m, 2H)

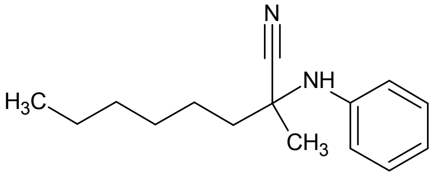

Entry 5. 2-methyl-2-(phenylamino)octanenitrile

CAS Registry Number: New Product.
^{1}H NMR (300 MHz; $CDCl_3$): δ = 0.90 (t, 3H), 1.33 (m, 7H), 1.55 (m, 3H), 1.64 (s, 3H), 3.69 (brs, 1H, NH), 6.91 (m, 3H), 7.26 (td, m, 2H)

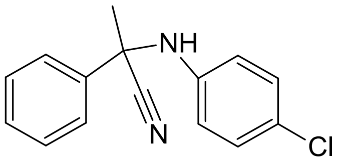

Entry 6. 2-((4-chlorophenyl)amino)-2-phenylpropanenitrile
CAS Registry Number: 81512-04-1.
^{1}H NMR (300 MHz; $CDCl_3$): δ = 1.94 (s, 3H), 4.30 (brs, 1H, NH), 6.47 (d, 2H), 7.06 (d, 2H), 7.40 (m, 3H), 7.59 (d, 2H).

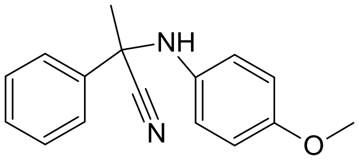

Table S4, entry 7. 2-((4-methoxyphenyl)amino)-2-phenylpropanenitrile [6]
CAS Registry Number: 81512-12-1.
^{1}H NMR (300 MHz; $CDCl_3$): δ = 2.02 (s, 3H), 3.71 (s,3H), 3.80 (brs, 1H, NH), 6.68 (m, 4H), 7.40 (m, 3H), 7.66 (m, 2H)

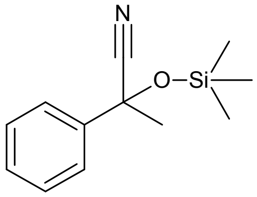

Entry 8b. 2-phenyl-2-((trimethylsilyl)oxy)propanenitrile
CAS Registry Number: 127462-20-8**.**
^{1}H NMR (300 MHz; $CDCl_3$): δ = 0.17 (s, 9H), 1.86 (s, 3H), 7.39 (m, 3H), 7.54 (m, 2H).

- **S7. SPECTRA FOR CHARACTERIZED COMPOUNDS**

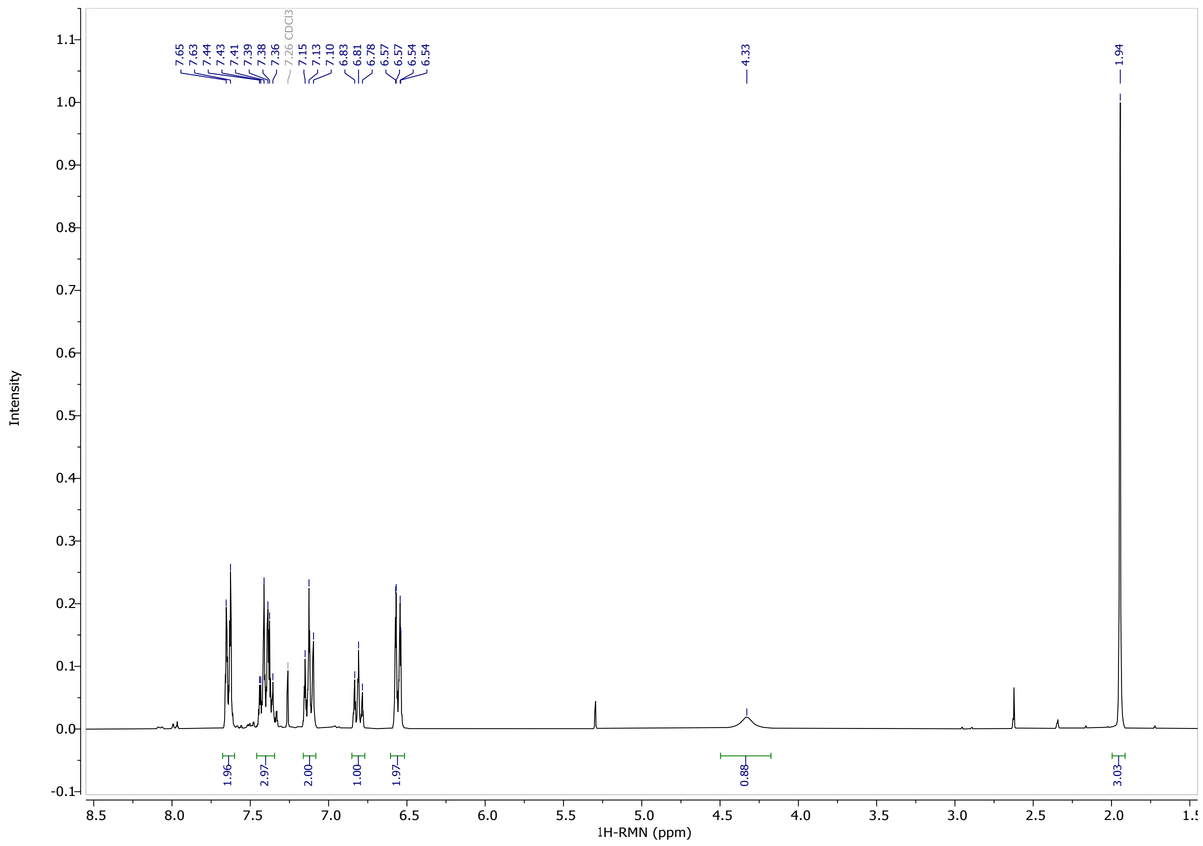

Figure S11. ^{1}H NMR spectra of 2-Phenyl-2-(phenylamino)propanenitrile. (Table S4, entry 1)

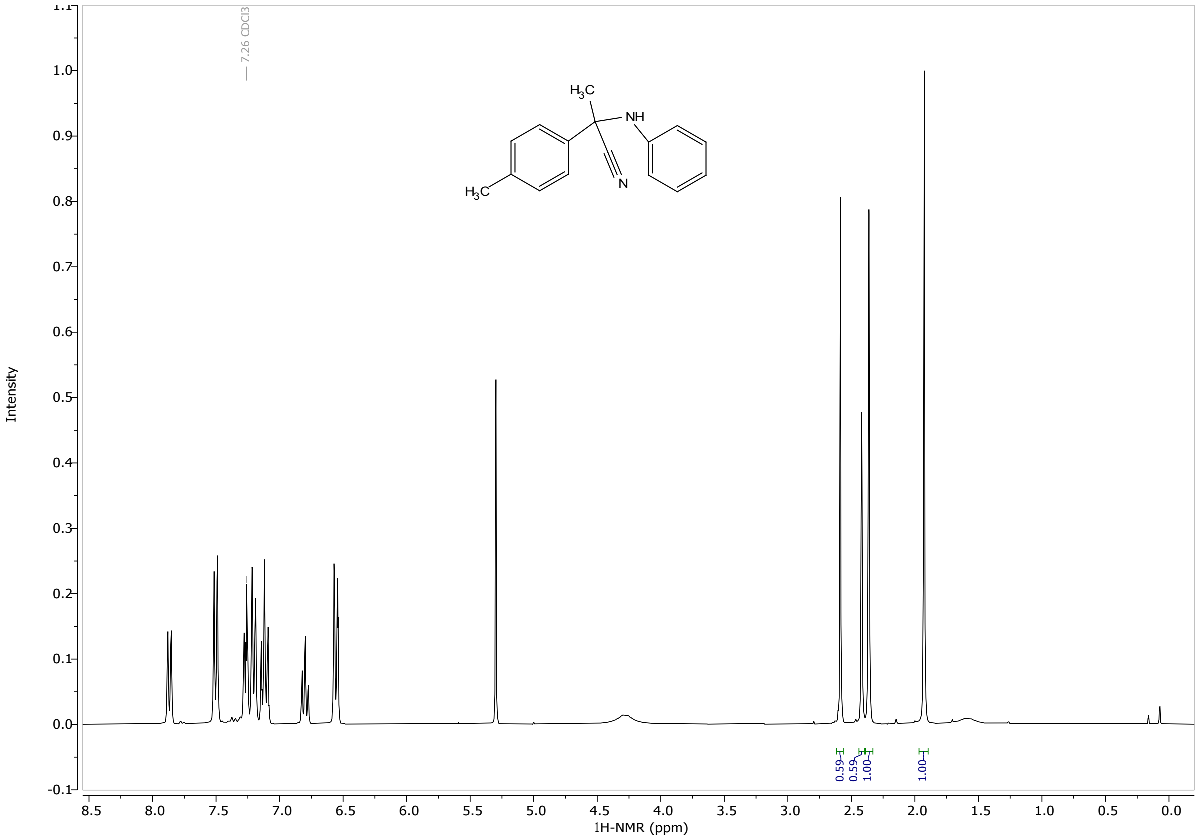

Figure S12. ^{1}H NMR spectra of 2-(phenylamino)-2-(*p*-tolyl)propanenitrile[6], (Table S4,entry 2).

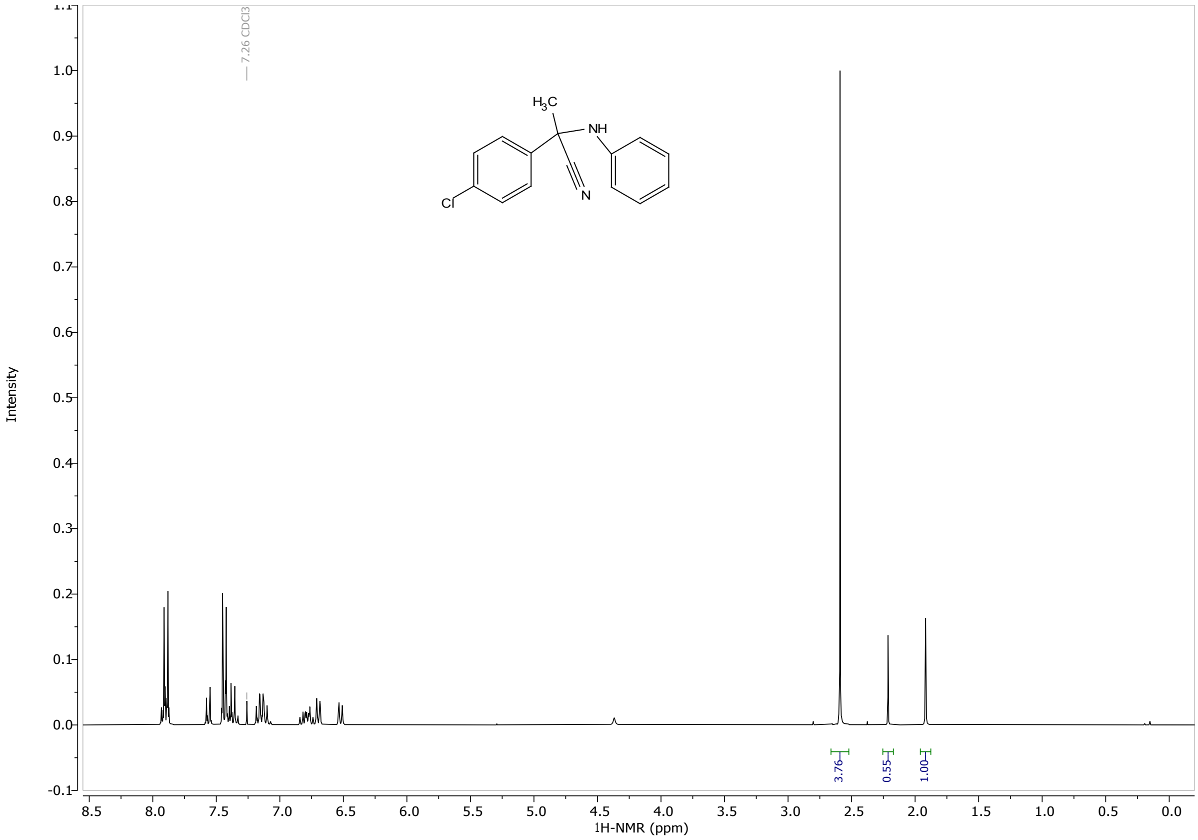

Figure S13. ^{1}H NMR spectra of 2-(4-chlorophenyl)-2-(phenylamino)propanenitrile [14],(Table S4, entry 3).

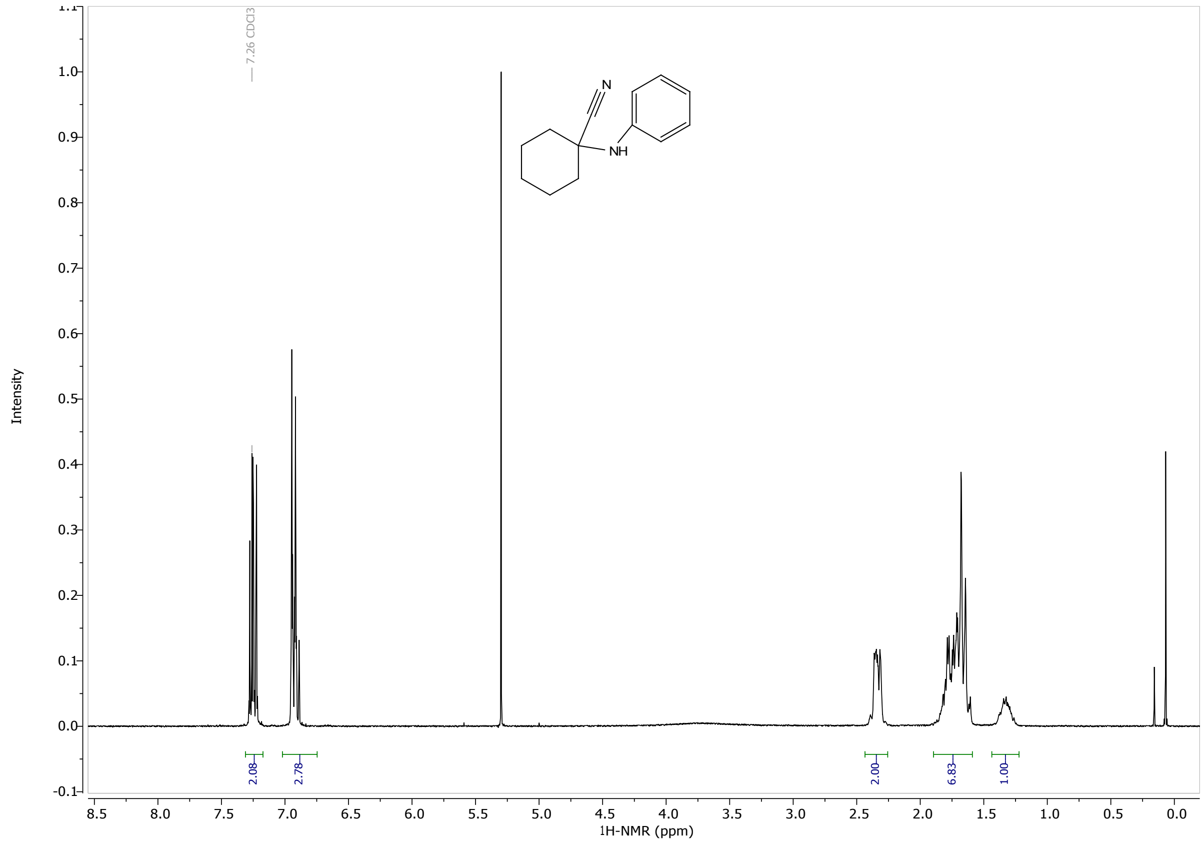

Figure S14. ^{1}H NMR spectra 1-(phenylamino)cyclohexanecarbonitrile [15], (Table S4, entry 4).

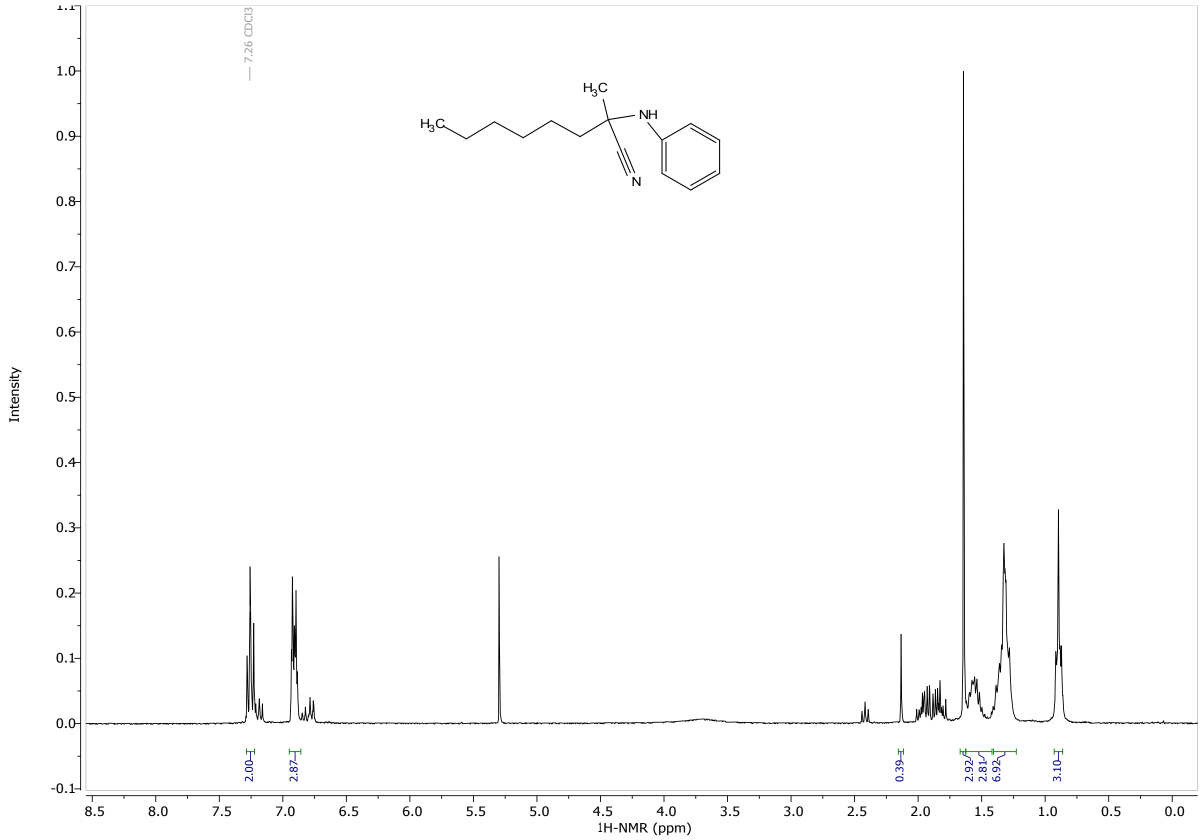

Figure S15. ^{1}H NMR spectra of 2-methyl-2-(phenylamino)octanenitrile,(Table S4, entry 5).

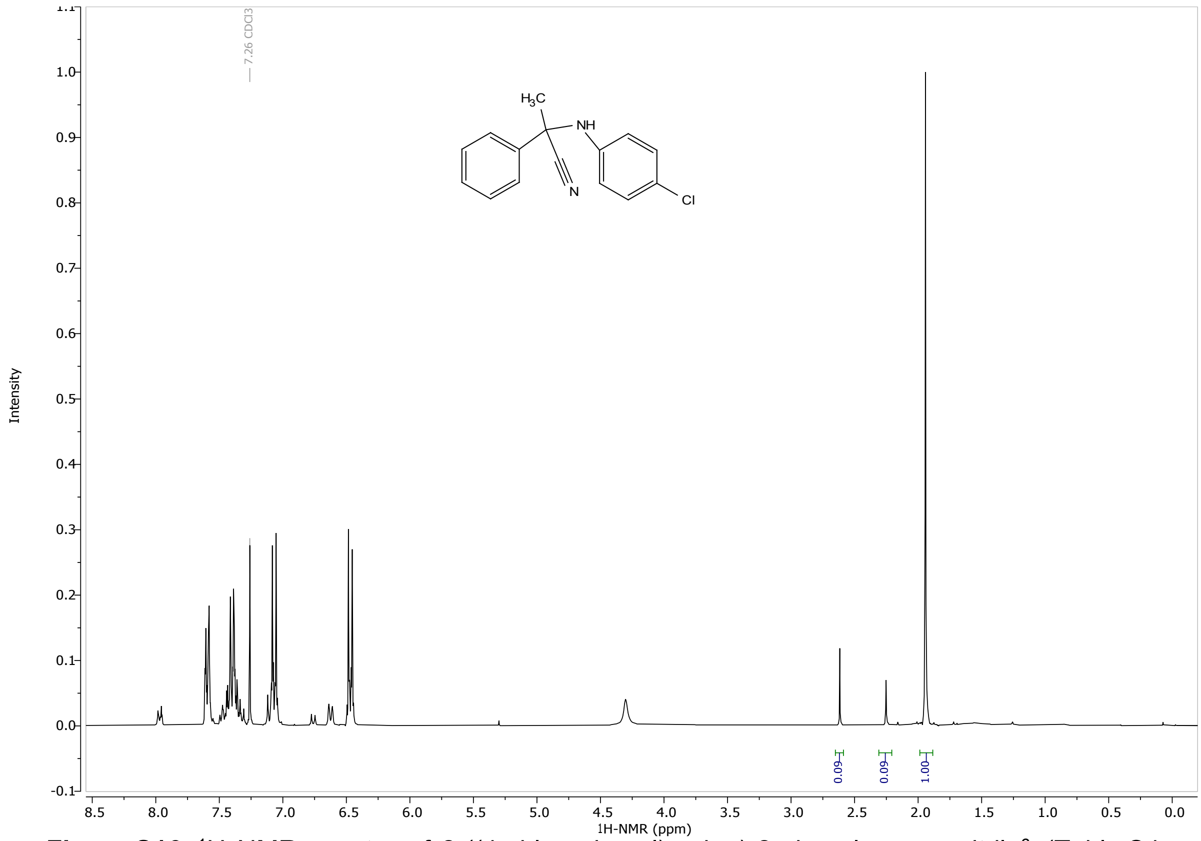

Figure S16. ^{1}H NMR spectra of 2-((4-chlorophenyl)amino)-2-phenylpropanenitrile[6], (Table S4, entry 6).

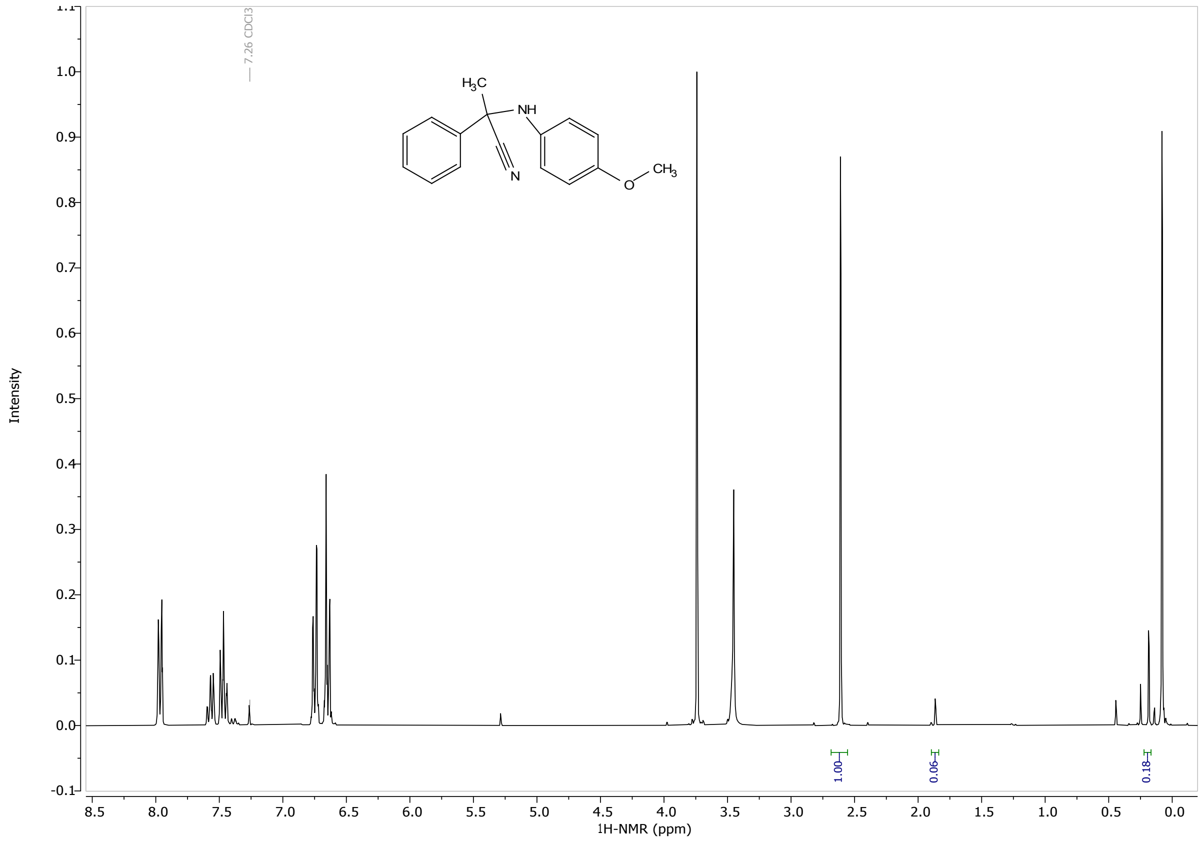

Figure S17. ^{1}H NMR spectra of 2-((4-methoxyphenyl)amino)-2-phenylpropanenitrile [6],(Table S4, entry 7).

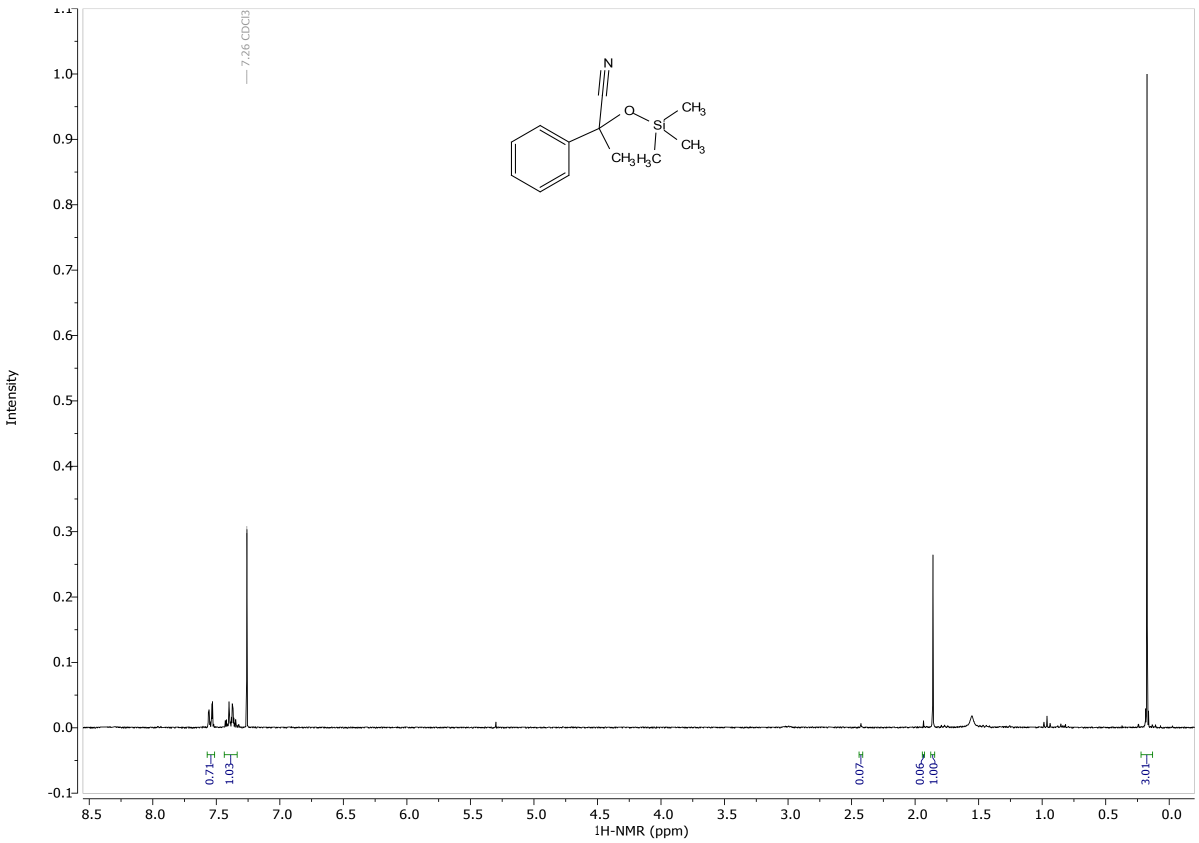

Figure S18. ¹H NMR spectra of 2-phenyl-2-((trimethylsilyl)oxy)propanenitrile, (Table S4, entry 8).

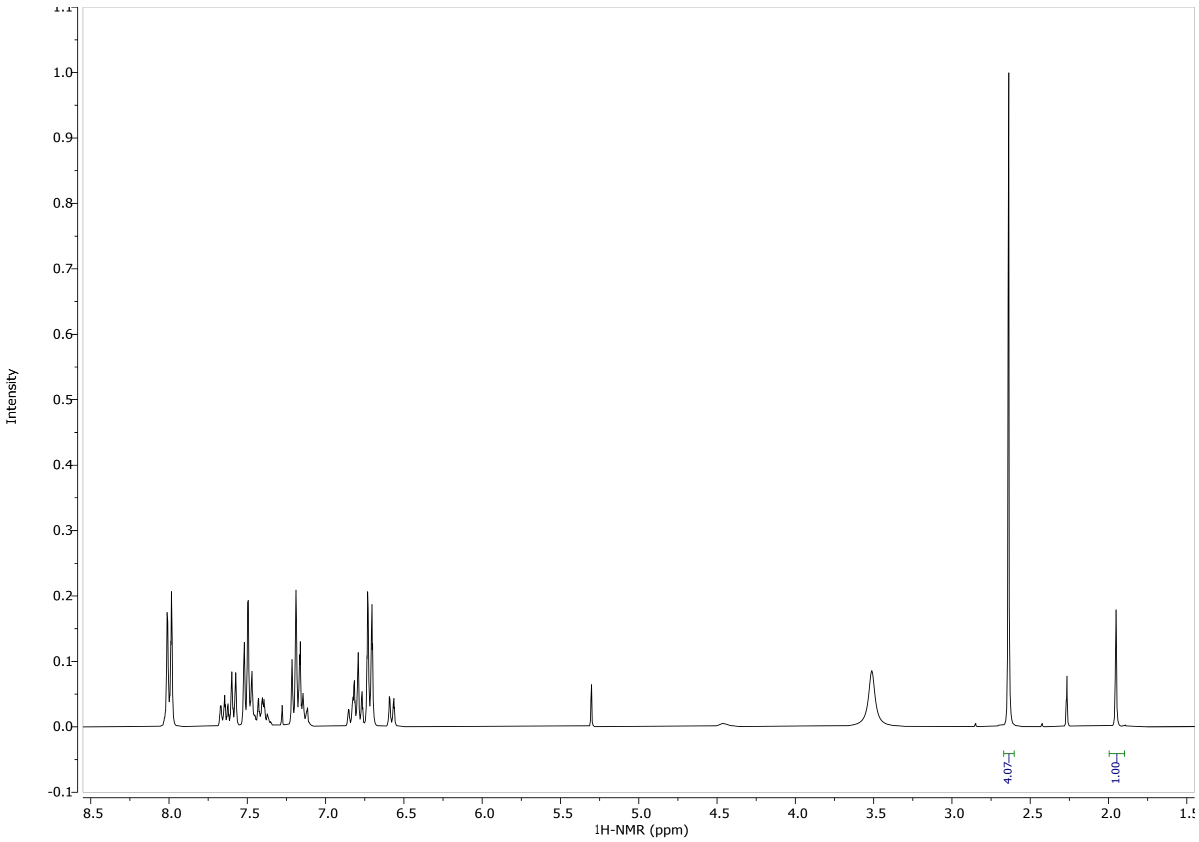

Figure S19. ¹H NMR spectra of 2-Phenyl-2-(phenylamino)propanenitrile. (Table S5, entry 1).

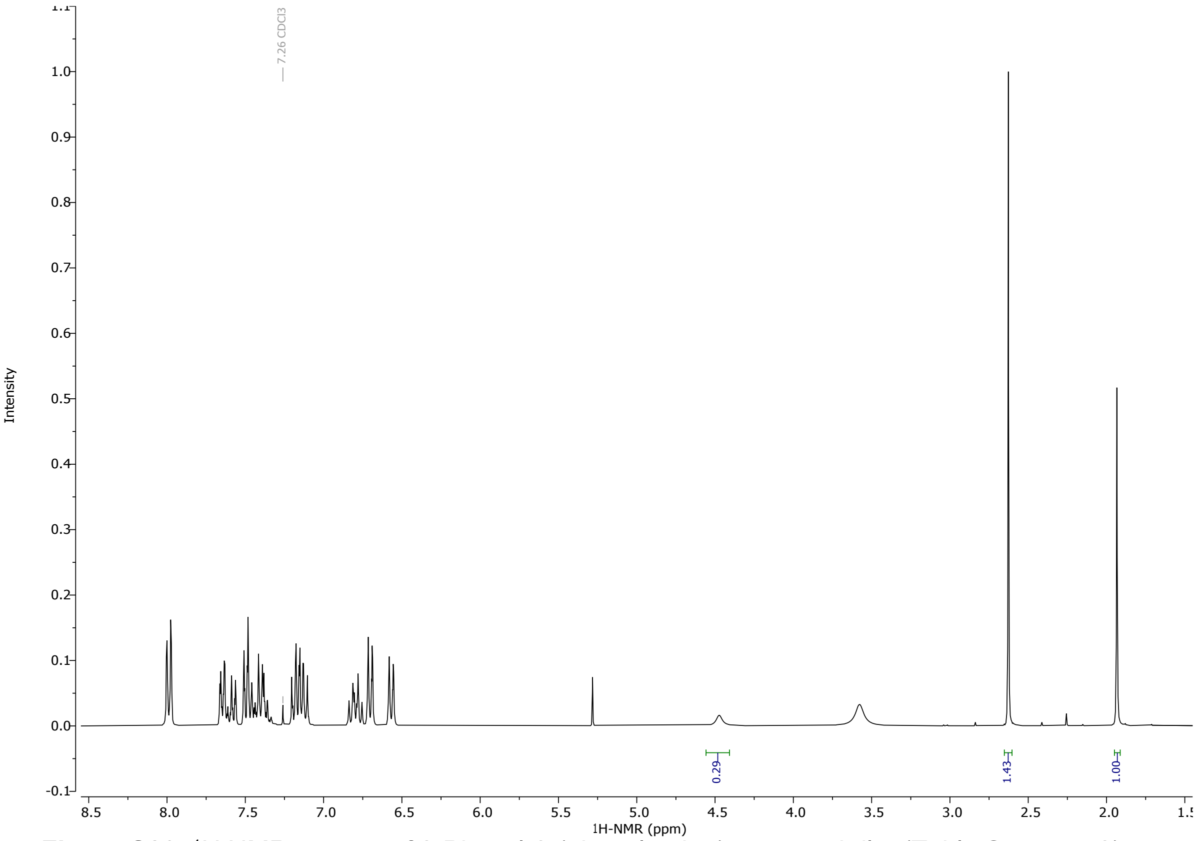

Figure S20. ^{1}H NMR spectra of 2-Phenyl-2-(phenylamino)propanenitrile. (Table S5, entry 2).

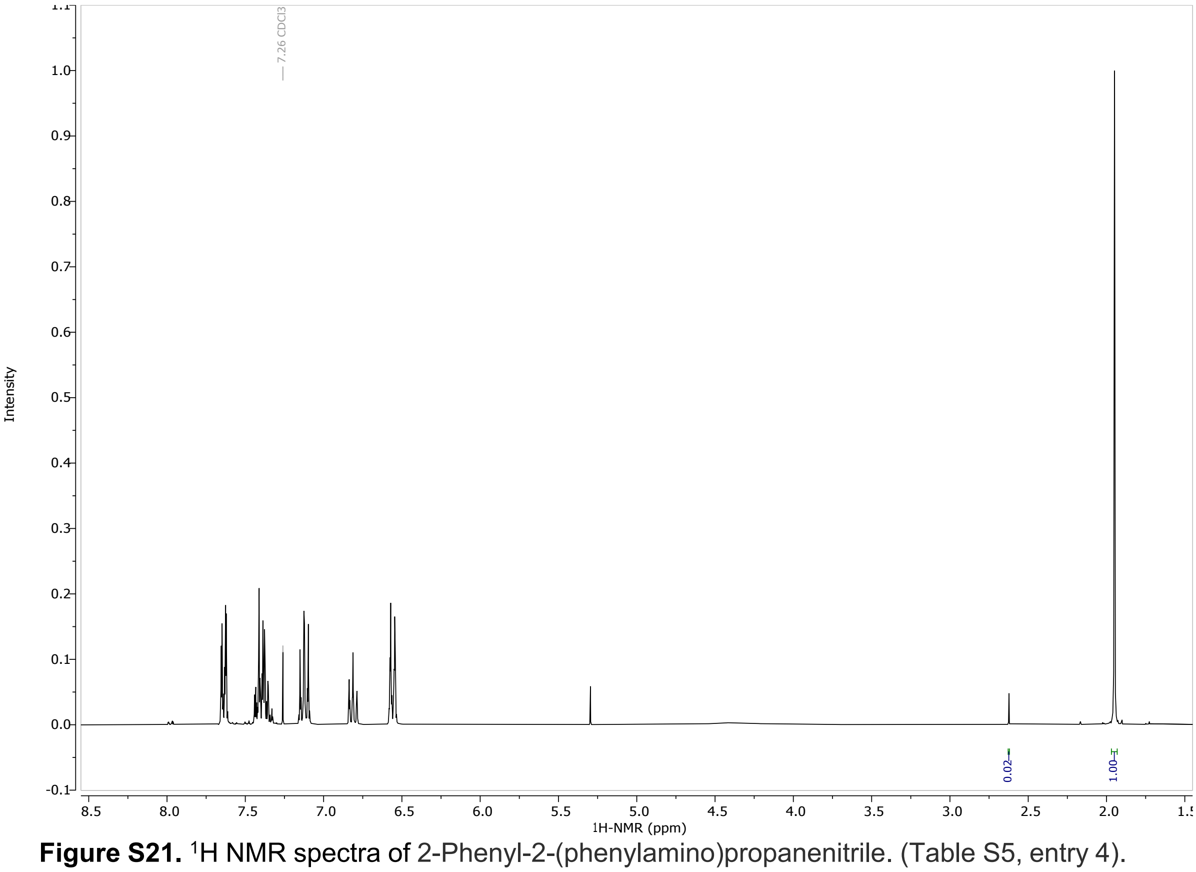

Figure S21. ^{1}H NMR spectra of 2-Phenyl-2-(phenylamino)propanenitrile. (Table S5, entry 4).

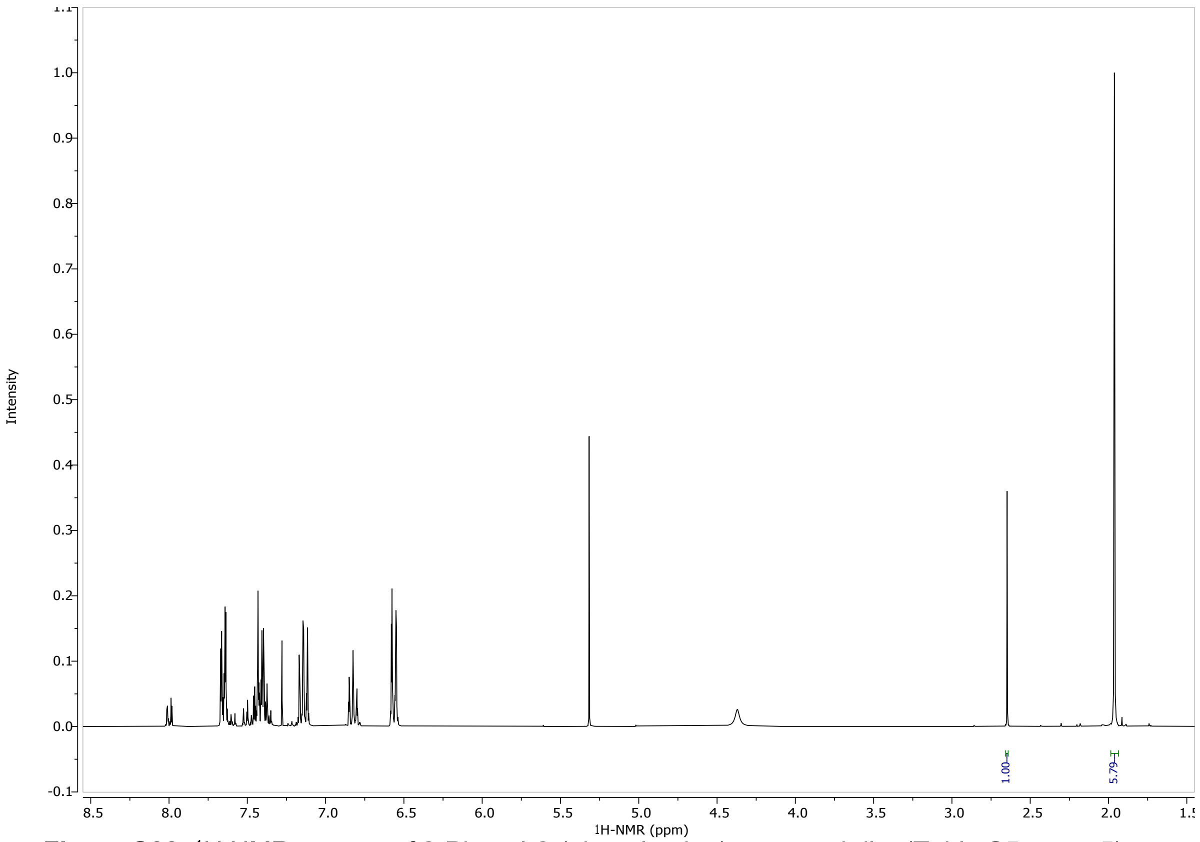

Figure S22. ¹H NMR spectra of 2-Phenyl-2-(phenylamino)propanenitrile. (Table S5, entry 5).

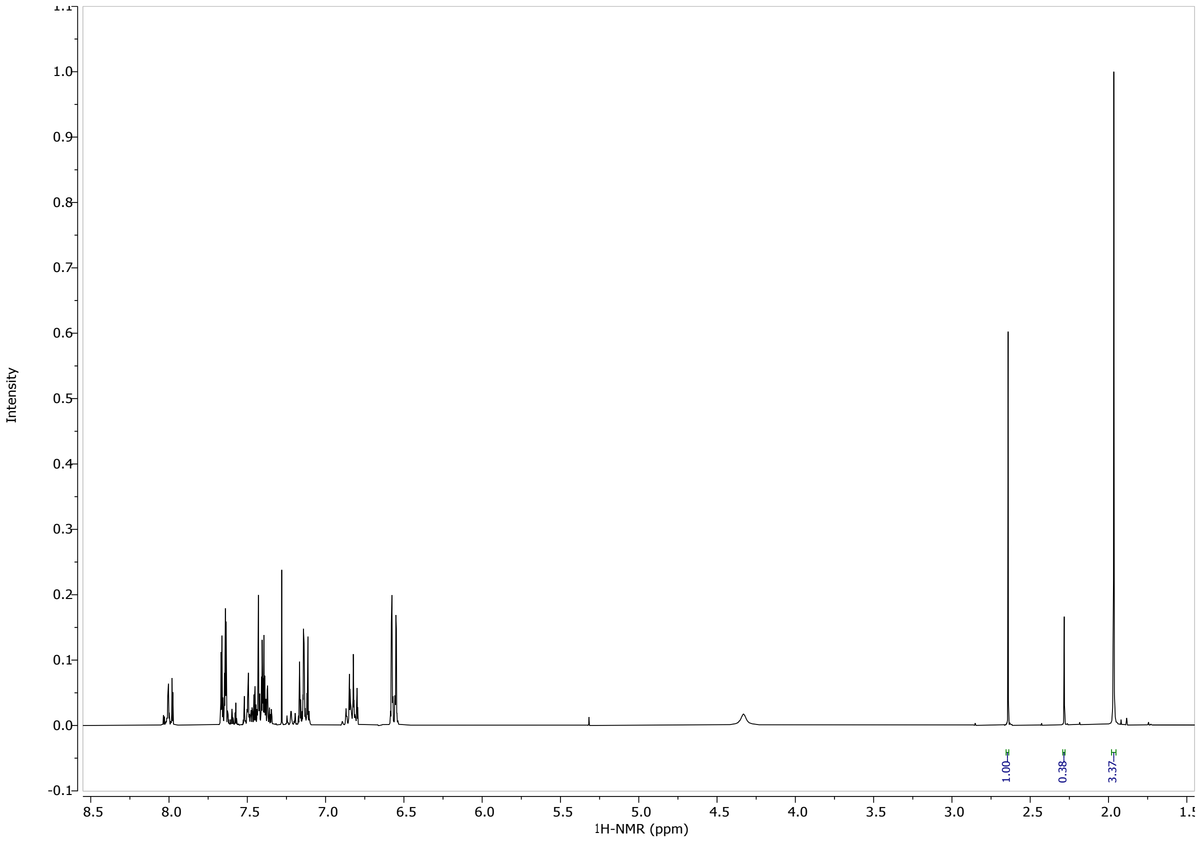

Figure S23. ¹H NMR spectra of 2-Phenyl-2-(phenylamino)propanenitrile. (Table S5, entry 6).

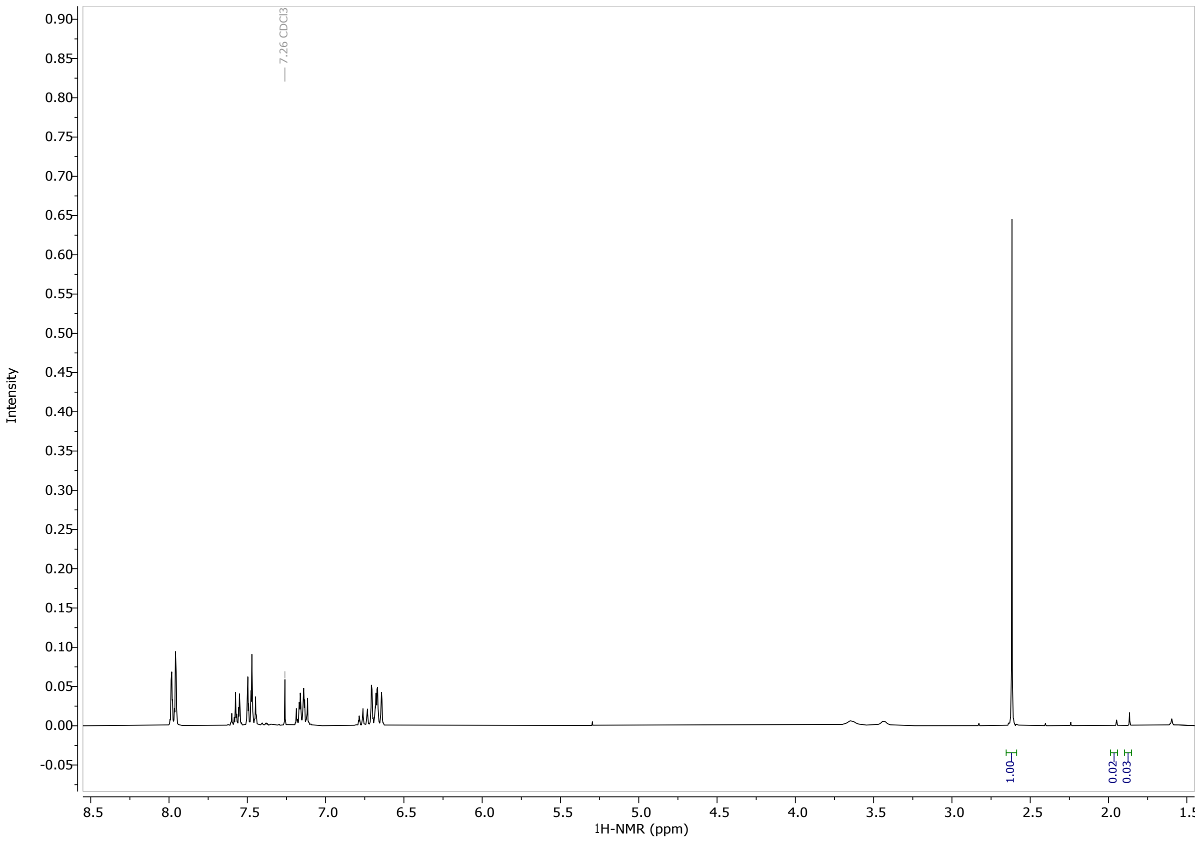

Figure S24. ^{1}H NMR spectra of 2-Phenyl-2-(phenylamino)propanenitrile. (Table S5, entry 7).

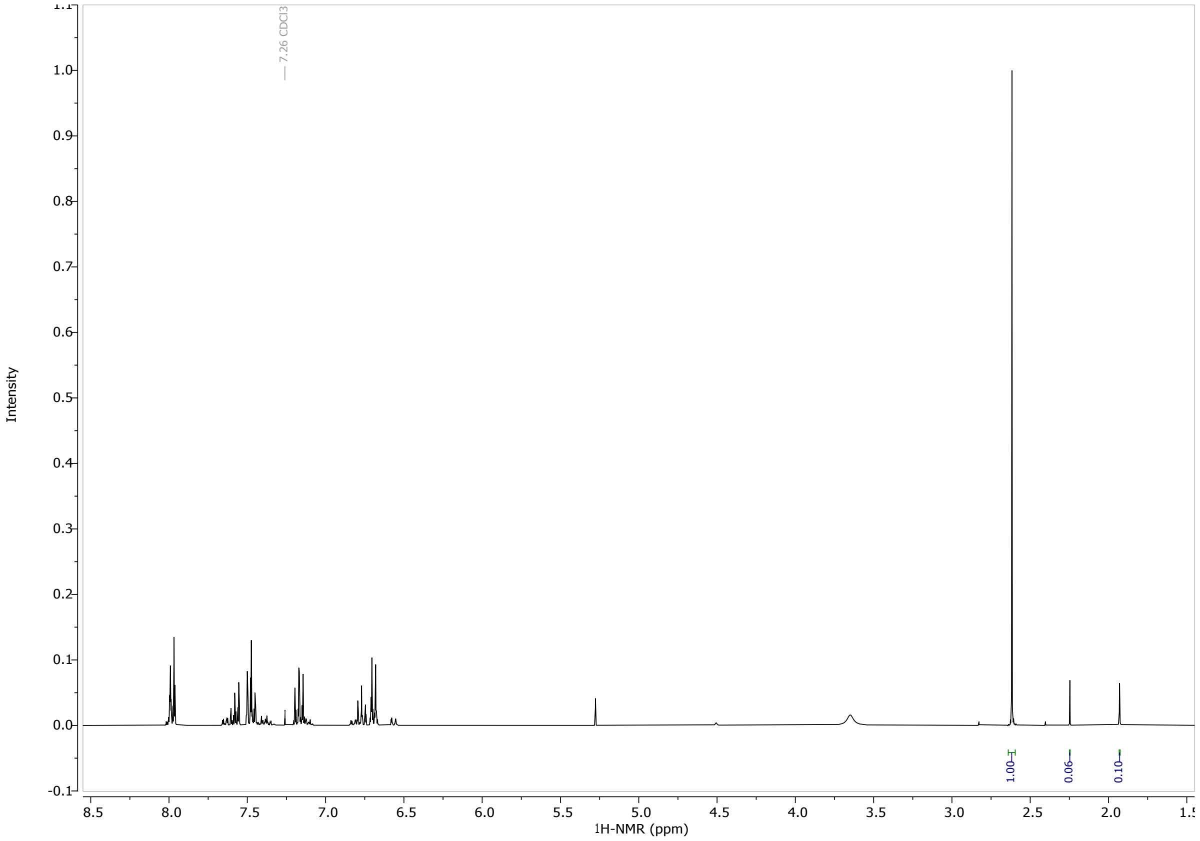

Figure S25. ^{1}H NMR spectra of 2-Phenyl-2-(phenylamino)propanenitrile. (Table S5, entry 8 (5min)).

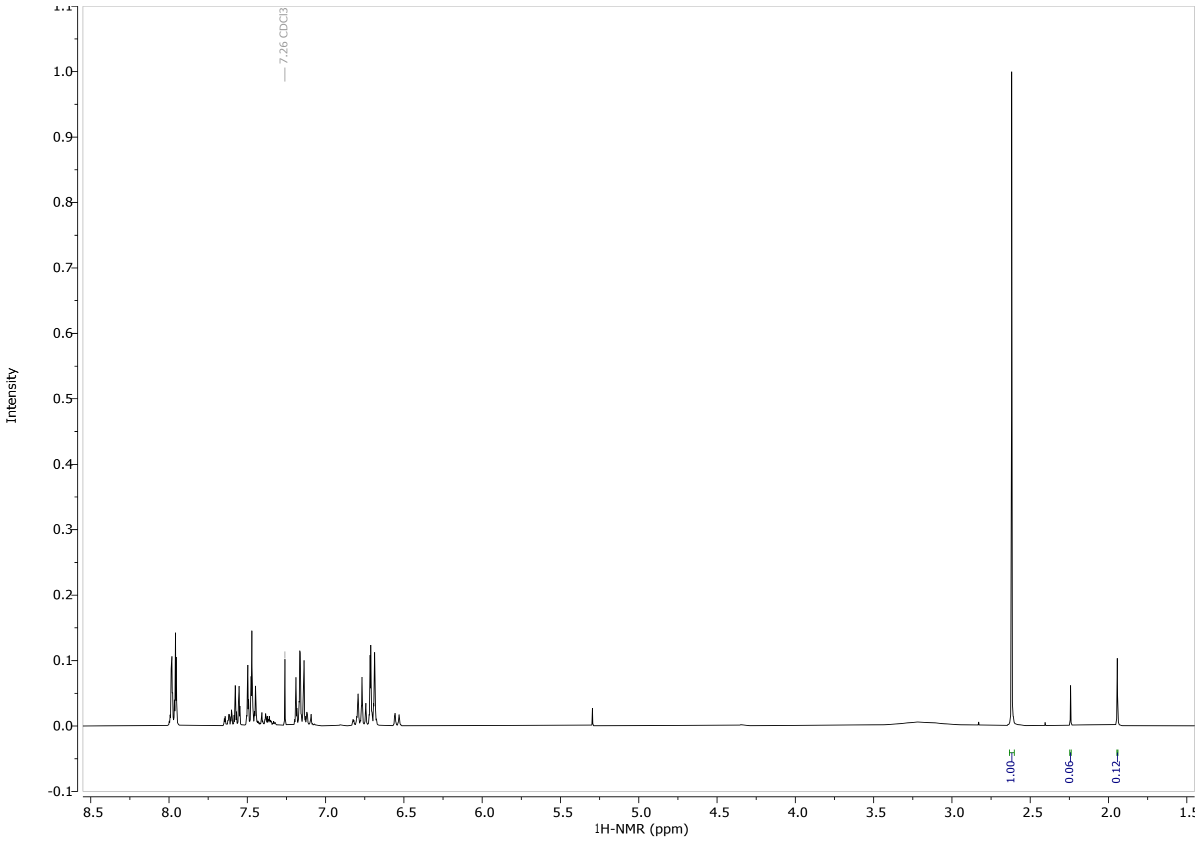

Figure S26. ^{1}H NMR spectra of 2-Phenyl-2-(phenylamino)propanenitrile.(Table S5, entry 8 (4h)).

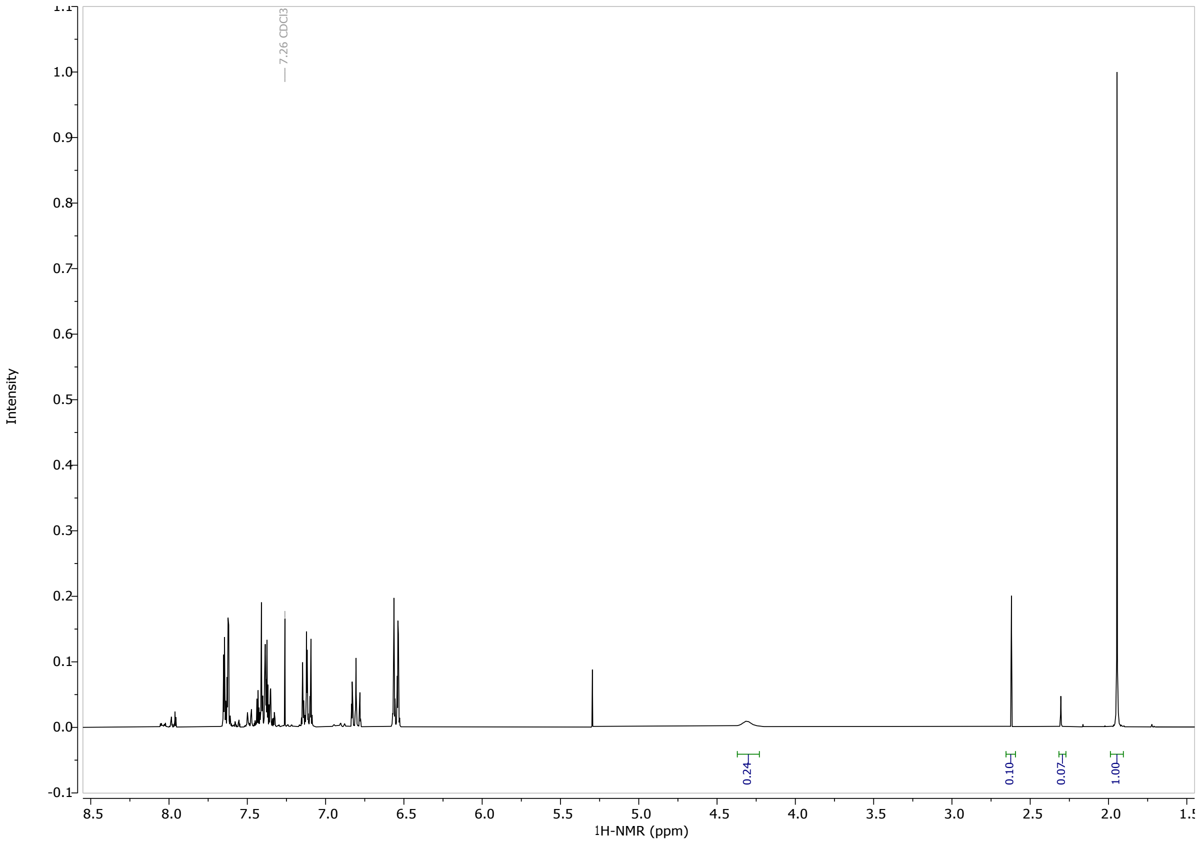

Figure S27. ^{1}H NMR spectra of 2-Phenyl-2-(phenylamino)propanenitrile. (Recycle Run 1).

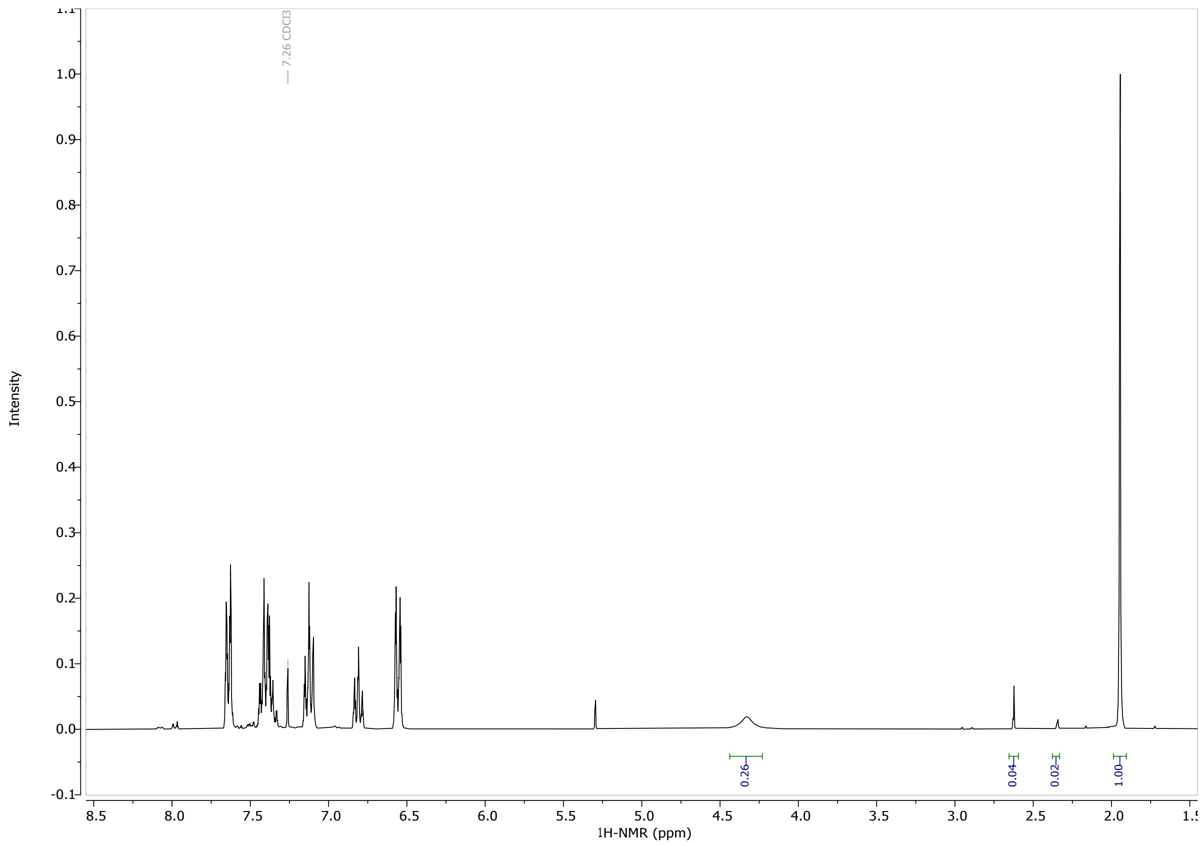

Figure S28. ^{1}H NMR spectra of 2-Phenyl-2-(phenylamino)propanenitrile. (Recycle Run 2).

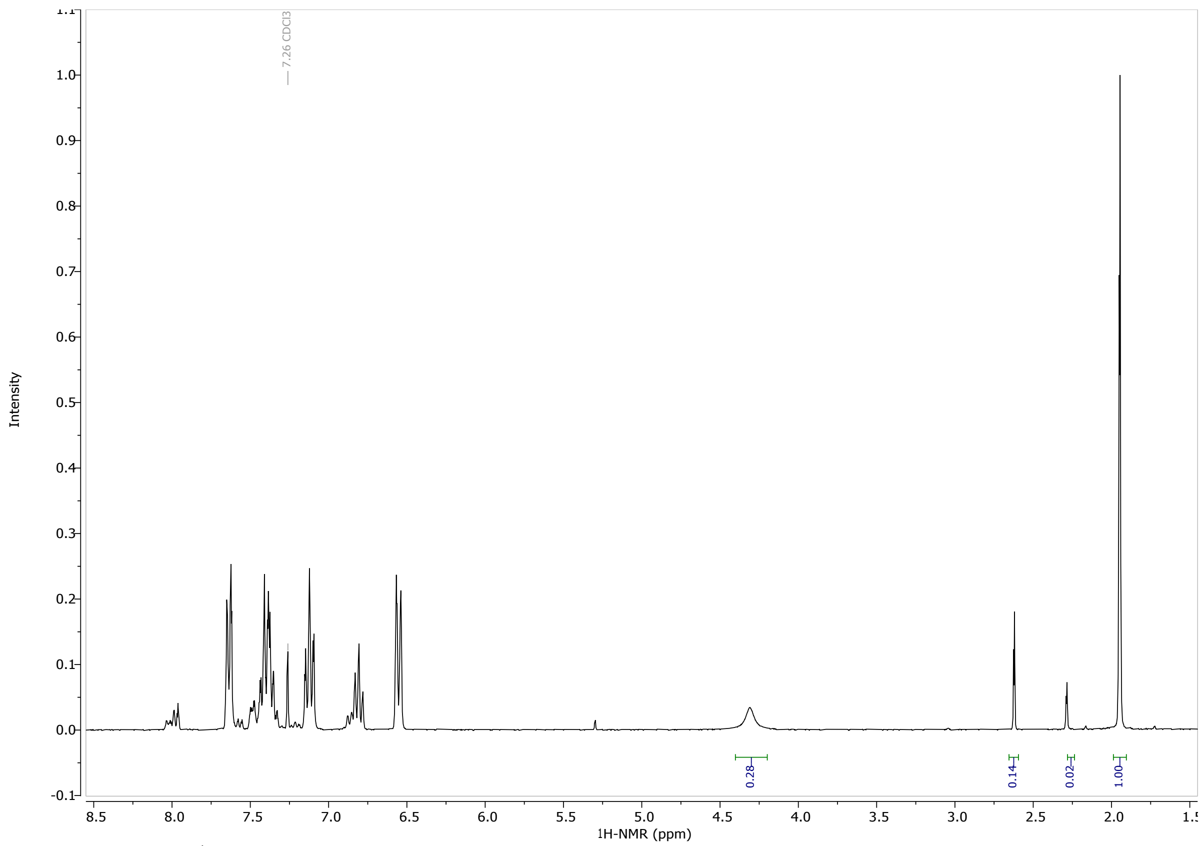

Figure S29. ^{1}H NMR spectra of 2-Phenyl-2-(phenylamino)propanenitrile. (Recycle Run 3).

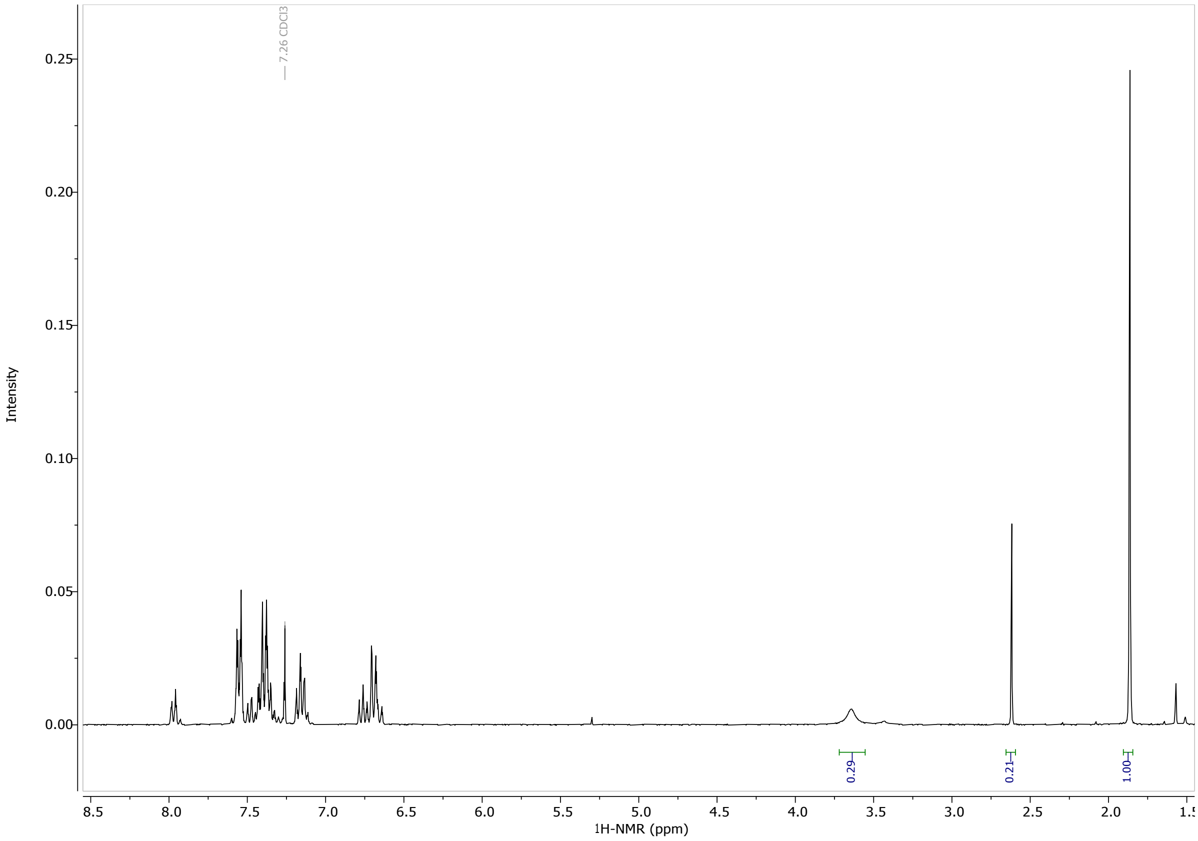

Figure S30. ^{1}H NMR spectra of 2-Phenyl-2-(phenylamino)propanenitrile. (Recycle Run 4).

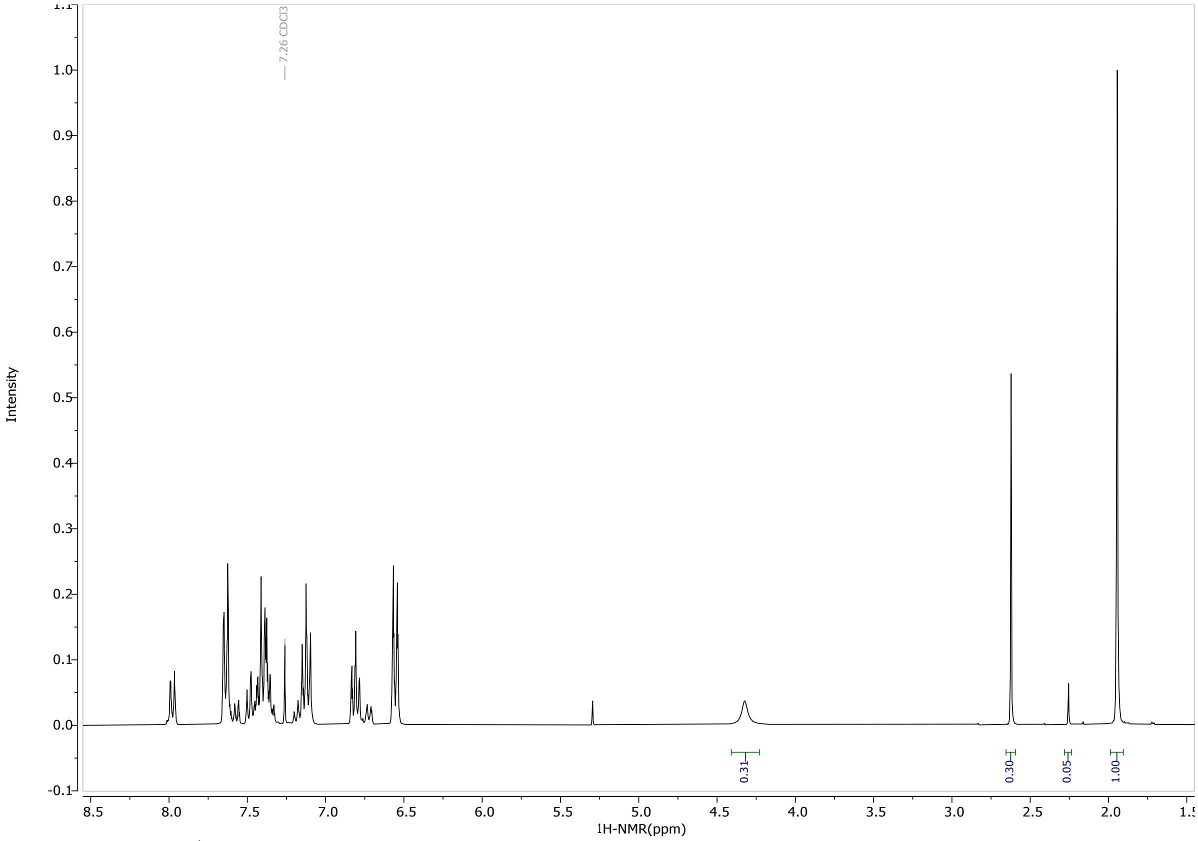

Figure S31. ^{1}H NMR spectra of 2-Phenyl-2-(phenylamino)propanenitrile. (Recycle Run 5).

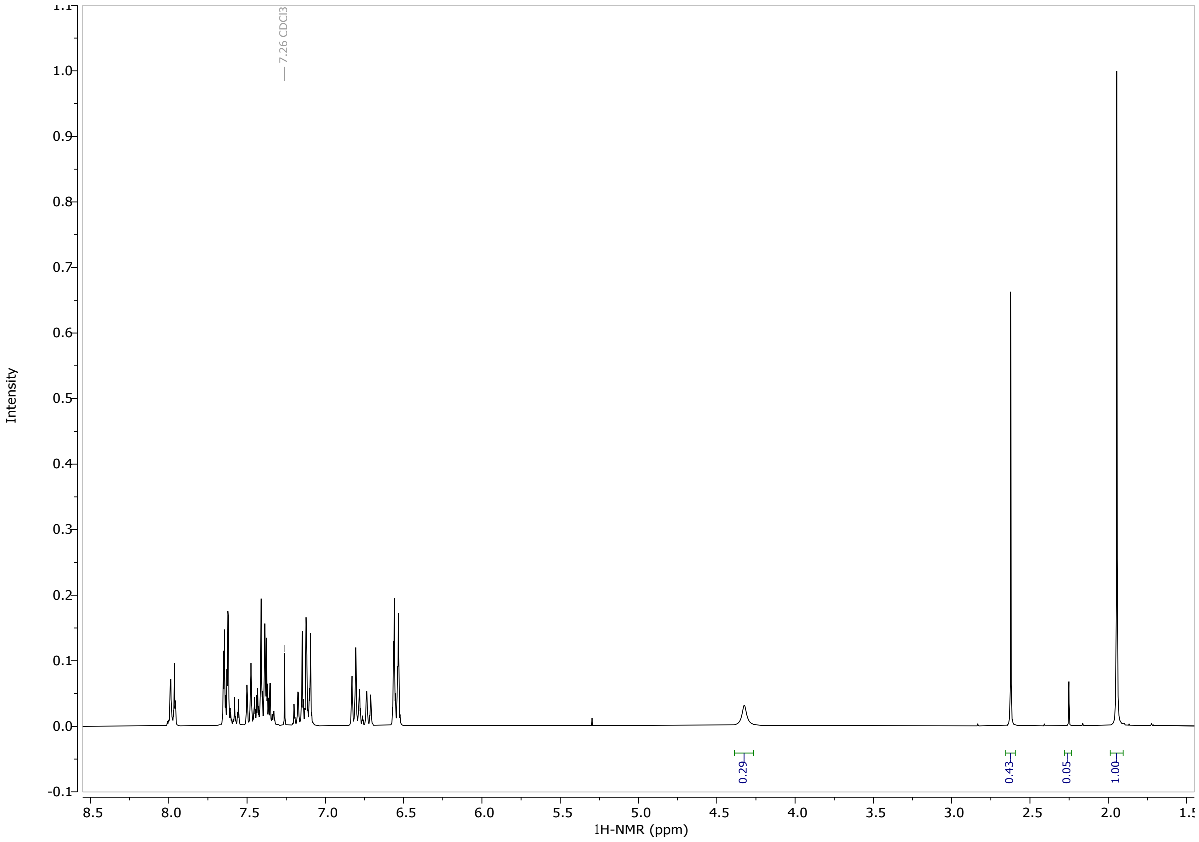

Figure S32. ^{1}H NMR spectra of 2-Phenyl-2-(phenylamino)propanenitrile. (Recycle Run 6).

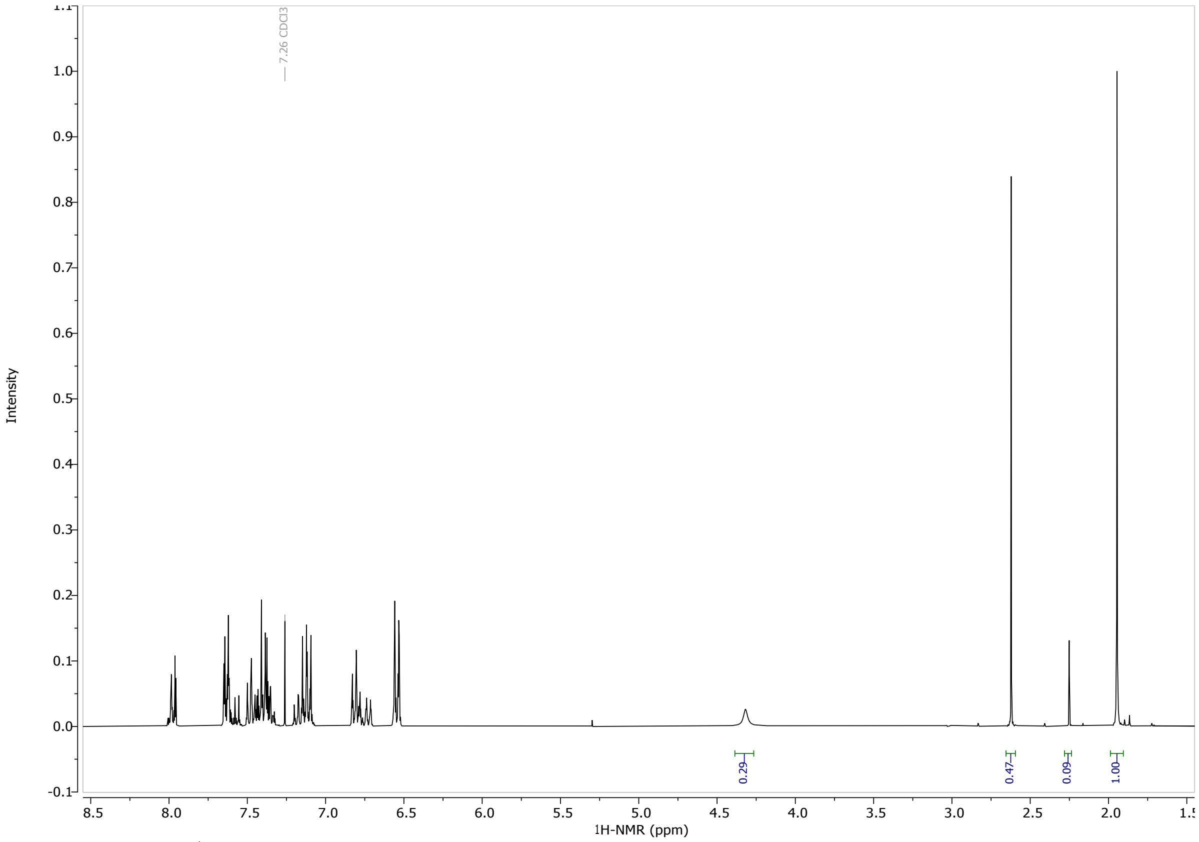

Figure S33. ^{1}H NMR spectra of 2-Phenyl-2-(phenylamino)propanenitrile. (Recycle Run 7).

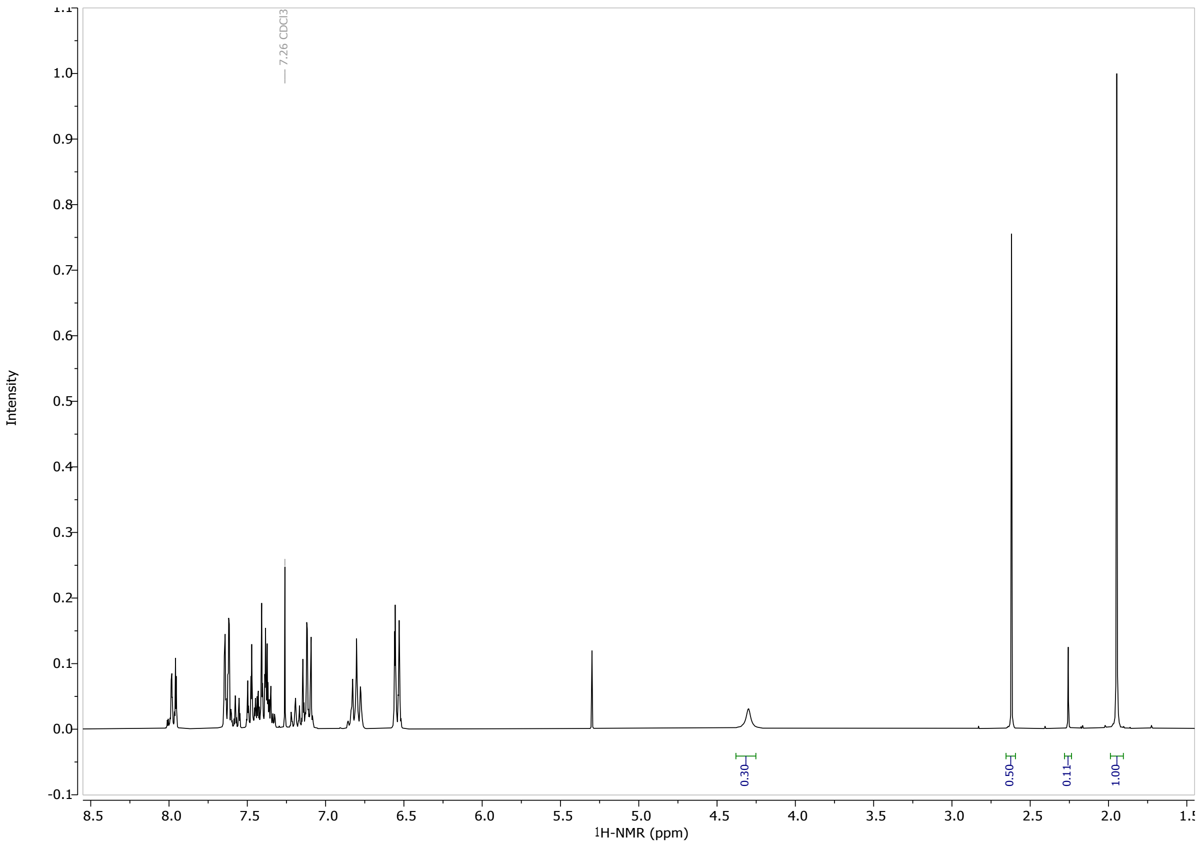

Figure S34 . ^{1}H NMR spectra of 2-Phenyl-2-(phenylamino)propanenitrile. (Recycle Run 8).

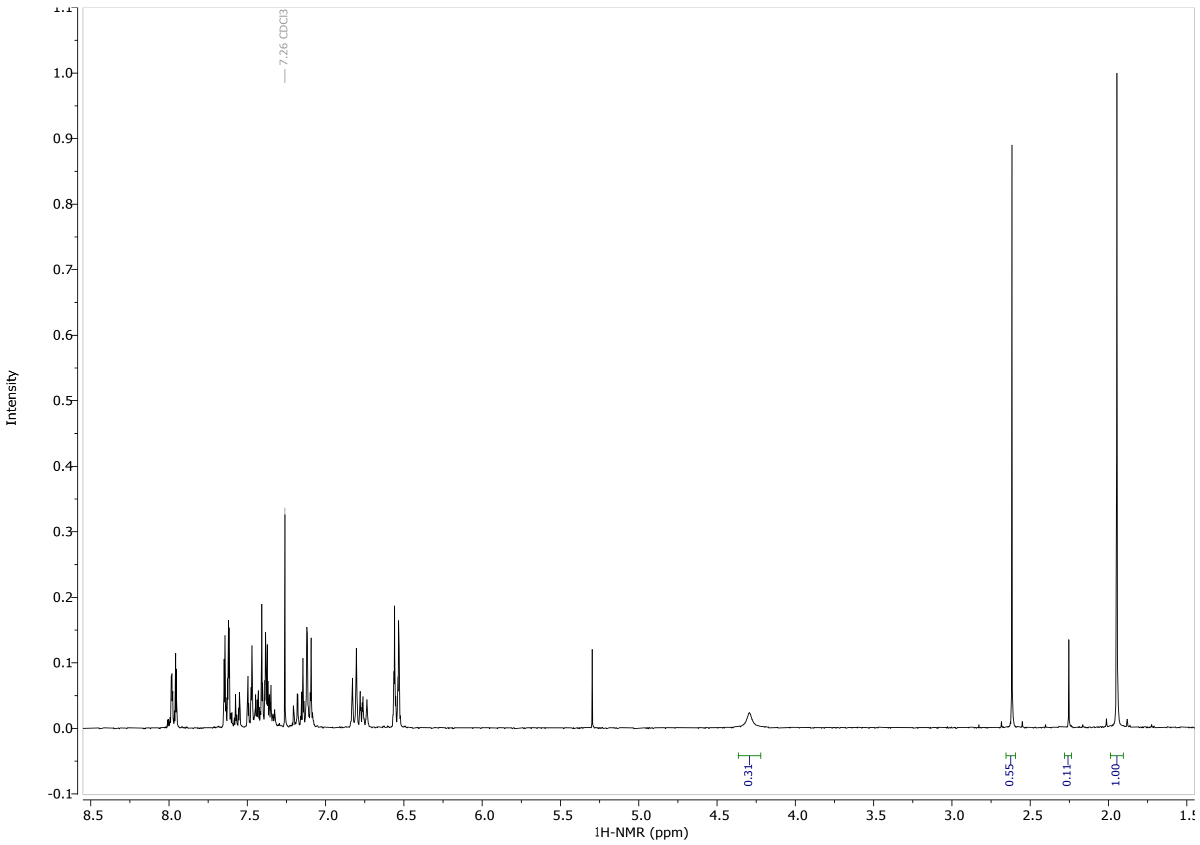

Figure S35. ^{1}H NMR spectra of 2-Phenyl-2-(phenylamino)propanenitrile. (Recycle Run 9)

- **S8. REFERENCES**

1 APEX2: Software Package. Bruker-AXS: Madison, WI, USA 2010.

2 Sheldrick, G. M. Shelxt-Integrated space-group and crystal structure determination. Acta. Cryst. 2015, A71, 3-8.

3 Dolomanov, O. V.; Bourhis, L. J.; Gildea, R. J.; Howard, J. A. K.; Puschmann, H., OLEX2: a complete structure solution, refinement and analysis programJ. Appl. Crystallogr. 2009, 42, 339-341.

4 Sheldrick, G. M., A short history of SHELX, Acta Crystallogr. Sect. A 2008, 64, 112-122

5 Inorganic Crystal Structure Database, ICSD.

6 Barbero, M.; Cadamuro, S.; Dughera, S.; Ghigo, G., o-Benzenedisulfonimide and its chiral derivative as Brønsted acids catalysts for one-pot three-component Strecker reaction. Synthetic and mechanistic aspects., Organic & Biomolecular Chemistry 2012, 10 (20), 4058-4068.

7 Karimi, B.; Zareyee, D., Solvent-free three component Strecker reaction of ketones using highly recyclable and hydrophobic sulfonic acid based nanoreactors, J. Mater. Chem. 2009, 19 (45), 8665-8670.

8 Bychinskaya, I.; Marinez, E. R.; Matthew, T.; Olah, G., Nafion–Fe: A New Efficient “Green” Lewis Acid Catalyst for the Ketonic Strecker Reaction Catalysis Letters. 2013, 143 (4), 303- 312.

9 Zhang, G.-W.; Zheng, D.-H.; Nie, J.; Wang, T.; Ma, J.-A., Brønsted acid-catalyzed efficient Strecker reaction of ketones, amines and trimethylsilyl cyanide, Organic & Biomolecular Chemistry 2010, 8 (6), 1399-1405.

10 Prakash, G. K. S.; Mathew, T.; Olah, G. A., Gallium(III) Triflate: An Efficient and a Sustainable Lewis Acid Catalyst for Organic Synthetic Transformations, Accounts Chem. Res. 2012, 45 (4), 565-577.

11 Hamachi, Y.; Katano, M.; Ogiwara, Y.; Sakai N. Production of Quaternary α-Aminonitriles by Means of Indium-Catalyzed Three-Component Reaction of Alkynes, Amines, and Trimethylsilyl Cyanide, Organic Letters 2016 18 (7), 1634-1637.

12 Xia, J.; Xu, J. N.; Fan, Y.; Song, T. Wang, Li.; Zheng, J. Y. NEXT, Indium Metal–Organic Frameworks as High-Performance Heterogeneous Catalysts for the Synthesis of Amino Acid Derivatives, Inorg. Chem. 2014, 53 (19), 10024-10026.

13 Aguirre-Díaz, L. M.; Gándara, F.; Iglesias, M.; Snejko, N.; Gutiérrez-Puebla, E.; Monge, M. Á., Tunable Catalytic Activity of Solid Solution Metal–Organic Frameworks in One-Pot Multicomponent Reactions , J. Am. Chem. Soc. 2015, 137, 19, 6132–6135

14 S. Sheik Mansoor, K. Aswin, K. Logaiya, S.P.N. Sudhan., An efficient one-pot three-component synthesis of α-amino nitriles via Strecker reaction catalysed by bismuth (III) nitrate, J. Saudi Chem. Soc., 2016, 20, S202-S210.

15 Shah, A. K.; Khan, N. H.; Sethia, G.; Saravanan, S.; Kureshy, R. I.; Abdi, S. H. R.; Bajaj, H.C. Highly Enantioselective Titanium-Catalyzed Cyanation of Imines at Room Temperature Applied Catalysis A: General 2012, 420, 22-30.

16 Pathare, S. P.; Akamanchi, K. G., Sulfated tungstate: a green catalyst for Strecker reaction, Tetrahedron Letters 2012, 53 (7), 871-875.
